# Symptomatology in 4-repeat tauopathies is associated with data-driven topology of [^18^F]-PI-2620 tau-PET signal

**DOI:** 10.1016/j.nicl.2023.103402

**Published:** 2023-04-11

**Authors:** Sonja Schönecker, Carla Palleis, Nicolai Franzmeier, Sabrina Katzdobler, Christian Ferschmann, Sebastian Schuster, Anika Finze, Maximilian Scheifele, Catharina Prix, Urban Fietzek, Endy Weidinger, Georg Nübling, Jonathan Vöglein, Marianne Patt, Henryk Barthel, Osama Sabri, Adrian Danek, Günter U. Höglinger, Matthias Brendel, Johannes Levin, for German Imaging Initiative for Tauopathies GII4T

**Affiliations:** a Department of Neurology, Ludwig-Maximilians-Universität München, LMU Munich, Munich, Germany; b German Center for Neurodegenerative Diseases (DZNE), Munich, Germany; c Munich Cluster for Systems Neurology (SyNergy), Munich, Germany; d Institute for Stroke and Dementia Research, Ludwig-Maximilians-Universität München, LMU München, Munich, Germany; e Department of Nuclear Medicine, Ludwig-Maximilians-Universität München, LMU Munich, Munich, Germany; f Department of Neurology and Clinical Neurophysiology, Schön Klinik München Schwabing, Munich, Germany; g Department of Nuclear Medicine, University Hospital of Leipzig, Leipzig, Germany; h European Reference Network for Rare Neurological Diseases (ERN-RND), Munich, Germany; i Department of Neurology, Hannover Medical School, Hannover, Germany

**Keywords:** Progressive supranuclear palsy, Corticobasal syndrome, Parkinsonism, 4-repeat tauopathy, [^18^F]PI-2620 tau-PET, Data-driven

## Abstract

In recent years in vivo visualization of tau deposits has become possible with various PET radiotracers. The tau tracer [^18^F]PI-2620 proved high affinity both to 3-repeat/4-repeat tau in Alzheimer’s disease as well as to 4-repeat tau in progressive supranuclear palsy (PSP) and corticobasal syndrome (CBS). However, to be clinically relevant, biomarkers should not only correlate with pathological changes but also with disease stage and progression. Therefore, we aimed to investigate the correlation between topology of [^18^F]PI-2620 uptake and symptomatology in 4-repeat tauopathies.

72 patients with possible or probable 4-repeat tauopathy, i.e. 31 patients with PSP-Richardson’s syndrome (PSP-RS), 30 with amyloid-negative CBS and 11 with PSP-non-RS/CBS, underwent [^18^F]PI-2620-PET. Principal component analysis was performed to identify groups of similar brain regions based on 20–40 min p.i. regional standardized uptake value ratio z-scores. Correlations between component scores and the items of the PSP Rating Scale were explored.

Motor signs like gait, arising from chair and postural instability showed a positive correlation with tracer uptake in mesial frontoparietal lobes and the medial superior frontal gyrus and adjacent anterior cingulate cortex. While the signs disorientation and bradyphrenia showed a positive correlation with tracer uptake in the parietooccipital junction, the signs disorientation and arising from chair were negatively correlated with tau-PET signal in the caudate nucleus and thalamus. Total PSP Rating Scale Score showed a trend towards a positive correlation with mesial frontoparietal lobes and a negative correlation with caudate nucleus and thalamus. While in CBS patients, the main finding was a negative correlation of tracer binding in the caudate nucleus and thalamus and a positive correlation of tracer binding in medial frontal cortex with gait and motor signs, in PSP-RS patients various correlations of clinical signs with tracer binding in specific cerebral regions could be detected.

Our data reveal [^18^F]PI-2620 tau-PET topology to correlate with symptomatology in 4-repeat tauopathies. Longitudinal studies will be needed to address whether a deterioration of signs and symptoms over time can be monitored by [^18^F]PI-2620 in 4-repeat tauopathies and whether [^18^F]PI-2620 may serve as a marker of disease progression in future therapeutic trials. The detected negative correlation of tracer binding in the caudate nucleus and thalamus with the signs disorientation and arising from chair may be due to an increasing atrophy in these regions leading to partial volume effects and a relative decrease of tracer uptake in the disease course. As cerebral regions correlating with symptomatology differ depending on the clinical phenotype, a precise knowledge of clinical signs and symptoms is necessary when interpreting [^18^F]PI-2620 PET results.

## Highlights

• In vivo visualization of tau deposits has become possibly by PET radiotracers.
• The tau tracer [^18^F]PI-2620 has high affinity to 4R-tau in 4-repeat tauopathies.
• PCA identified 13 cerebral regions based on tau-PET SUVr z-scores in PSP and CBS.
• Tau-PET topology correlates with symptomatology in 4-repeat tauopathies.
• A precise knowledge of clinical signs is necessary when interpreting tau-PET.

## 1 Introduction

Tauopathies comprise a number of adult-onset neurodegenerative diseases characterized by intracellular aggregation and transcellular propagation of abnormal forms of the microtubule-associated protein tau (MAPT), commonly known as tau protein (Kovacs, 2015). Depending on whether tau deposits predominantly contain isoforms with three or four repeats of the microtubule-binding domain, three-repeat- (3R), four-repeat- (4R) and mixed 3R/4R-tauopathies (Rösler et al., 2019) can be differentiated. According to the appearance, histologic localization, and anatomical distribution of the 4R-tau aggregates, 4R-tauopathies are classified into progressive supranuclear palsy (PSP), corticobasal degeneration (CBD), argyrophilic grain disease, globular glial tauopathy and frontotemporal lobar degeneration due to *MAPT* mutations with 4R-tau pathology (Kovacs, 2015). The clinical spectrum of 4R-tauopathies is remarkably heterogenous. While PSP Richardson’s syndrome (RS) and corticobasal syndrome (CBS) are the most common phenotypes associated with 4R-tauopathies, other syndromes like behavioural variant frontotemporal dementia, non-fluent/agrammatic variant of primary progressive aphasia, apraxia of speech, PSP with parkinsonism and PSP with progressive gait freezing can also be caused by 4R-tauopathies (Respondek et al., 2014, Respondek et al., 2017). Due to the heterogeneity of symptomatology within the 4R-tauopathies but also because of phenotypic overlap with neurodegenerative diseases caused by differing protein aggregates, like amyloid β and 3R- and 4R-tau in Alzheimer’s disease (AD) or TAR DNA binding protein 43 (TDP-43) proteinopathies, a precise antemortem prediction of the underlying pathology is difficult based on clinical features alone. In vivo visualization of tau deposits has become possible with various radiotracers for use with positron emission tomography (PET). While first-generation tau tracers suffered from substantial off-target binding, the next generation tau tracer [^18^F]PI-2620 proved absent off-target binding to monoamine oxidases, high affinity to 3R/4R tau in AD and also revealed binding in the 4R tauopathies PSP (Brendel et al., 2020, Franzmeier et al., 2022) and CBS (Franzmeier et al., 2022, Palleis et al., 2021). However, in order to be clinically relevant, biomarkers should not only correlate with pathological changes but also with disease stage and progression which can be utilized to evaluate therapeutic responses (Frank and Hargreaves, 2003). Our previous studies with [^18^F]PI-2620 in PSP (Brendel et al., 2020) and CBS (Palleis et al., 2021) indicated only a weak association with clinical symptom severity but we hypothesized that the lacking associations could be related to limited regions of interest and global severity scores that did not take underlying topology into account. Therefore, we aimed to investigate the correlation between the intensity of [^18^F]PI-2620 uptake in a data-driven selection of supratentorial brain regions and disease severity measured by specific items of the PSP Rating Scale (PSPRS) in patients with possible or probable 4R-tauopathies.

## 2 Materials and methods

### 2.1 Ethics statement

The study was performed according to the Declaration of Helsinki (1991). PET data analysis was approved by the local ethics committee (Medical Faculty, Ludwig-Maximilians-Universität München, Munich, Germany, project numbers 17–569 and 19–022). Written informed consent was obtained from every participant.

The CBS study cohort is embedded in “Activity of Cerebral Networks, Amyloid and Microglia in Aging and Alzheimer’s Disease (ActiGliA)”, a prospective cohort study at Ludwig-Maximilians-Universität München (LMU), Munich, Germany, approved by the local ethics committee (project number 17–755).

### 2.2 Clinical evaluation

We enrolled 72 patients from the outpatient clinic for neurodegenerative diseases at the Department of Neurology, Ludwig-Maximilians-Universität München, Munich, Germany with a diagnosis of possible or probable 4R-tauopathy according to current diagnostic criteria (Hoglinger et al., 2017, Armstrong et al., 2013). 31 have been diagnosed with PSP-Richardson’s syndrome (PSP-RS), 30 with CBS and 11 with PSP-non-RS/CBS (5 with PSP-parkinsonism, 3 with PSP-frontotemporal dementia, 2 with PSP-speech/language disorders and 1 with PSP-progressive gait freezing). Tau-PET and clinical data of PSP (Brendel et al., 2020) and CBS (Palleis et al., 2021) patients were previously published. As only patients seen and treated at the at the Department of Neurology, Luwdig-Maximilians-Universität, München, Munich, Germany were included in the current analysis, sample sizes differ slightly compared to previously published data. Clinical diagnosis of CBS was established according to the MDS-PSP criteria (Hoglinger et al., 2017). All CBS patients additionally fulfilled the Armstrong criteria of probable or possible CBD-CBS (Armstrong et al., 2013). In CBS patients, primary AD pathology was excluded by evaluation of β-amyloid status in cerebrospinal fluid (Aβ42 concentration and Aβ42/40 ratio) and/or [^18^F]flutemetamol and [^18^F]florbetaben PET respectively. The threshold for Aβ42/40 ratio was set at < 5.5% and the threshold for Aβ42 concentration was set at < 375 pg/ml according to standardized laboratory diagnostics at Ludwig-Maximilians-Universität München, Munich, Germany. The control group used for generation of regional tau-PET z-scores consisted of 14 age-matched healthy subjects whose demographic data are provided elsewhere (Palleis et al., 2021). Disease severity was measured with the PSP Rating Scale (PSPRS; 28 items, score range 0–100, with higher numbers indicating higher severity) (Golbe and Ohman-Strickland, 2007) while functional independence was measured using the Schwab and England Activities of Daily Living scale (SEADL). Cognitive screening was performed by the Montreal Cognitive Assessment (MoCA) scale. Additionally, age, education and disease duration, i.e. subjective symptom onset to performance of PI-2620 tau-PET, were assessed. Demographics of the study sample are provided in Table 1.

**Table 1:** Demographic and clinical data of the study sample.

	PSP-RS	CBS	PSP-non-RS/CBS	*p*-value
	n = 31	N = 30	n = 11	
Sex (m/f)	17/14	14/16	8/3	0.33
Age (y)	71.8 ± 8.3	68.2 ± 8.5	71.6 ± 5.4	0.18
Education (y)	11.8 ± 3.2	12.2 ± 2.5	12.4 ± 2.5	0.81
Disease duration (mo)	41.8 ± 42.7	33.2 ± 18.5	42.4 ± 33.6	0.55
MoCA	22.7 ± 4.7	23.2 ± 4.7	20.8 ± 3.4	0.36
SEADL	56.7 ± 19.4	63.3 ± 17.7	60.0 ± 20.5	0.40
PSPRS total score	35.2 ± 11.9^b^	27.2 ± 13.8^a^	28.5 ± 9.4	**0.04**
**PSPRS sub-items**				
Withdrawal	0.81 ± 0.54	0.47 ± 0.57	0.91 ± 0.54	**0.02**
Irritability	0.16 ± 0.37	0.30 ± 0.60	0.27 ± 0.65	0.42
Dysphagia for solids	0.58 ± 0.50^b^	0.20 ± 0.41^a^	0.18 ± 0.40	**<0.01**
Using knife and fork, buttoning clothes, washing hands and face	1.71 ± 1.04	1.67 ± 0.84	1.00 ± 0.89	0.88
Falls	1.84 ± 1.04^b^	0.93 ± 1.11^a^	1.36 ± 1.43	**<0.01**
Urinary incontinence	0.77 ± 1.12	0.57 ± 1.01	0.82 ± 0.87	0.45
Sleep difficulty	0.84 ± 0.97	0.53 ± 0.90	0.73 ± 1.01	0.11
Disorientation	0.71 ± 0.78	0.57 ± 0.86	1.36 ± 1.21	0.30
Bradyphrenia	1.45 ± 0.93	1.20 ± 1.21	1.64 ± 1.03	0.18
Emotional incontinence	0.84 ± 1.13	0.60 ± 1.00	0.91 ± 1.38	0.28
Grasping/imitative/utilizing behaviour	0.94 ± 0.85	1.20 ± 1.13	0.91 ± 0.83	0.44
Dysarthria	1.71 ± 0.90	1.33 ± 1.27	1.55 ± 0.93	0.16
Dysphagia	1.19 ± 0.98^b^	0.43 ± 0.90^a^	0.45 ± 0.69	**<0.01**
Ocular voluntary upward command movement	3.10 ± 1.11^b, c^	1.30 ± 1.39^a^	1.64 ± 1.21^a^	**<0.01**
Ocular voluntary downward command movement	2.29 ± 1.35^b, c^	0.77 ± 1.04^a^	0.82 ± 0.87^a^	**<0.01**
Ocular voluntary left and right command movement	1.39 ± 1.05^b, c^	0.57 ± 0.82^a^	0.55 ± 0.52^a^	**<0.01**
Eyelid dysfunction	1.26 ± 0.82^b^	0.60 ± 0.50^a^	0.91 ± 0.54	**<0.01**
Limb rigidity	1.23 ± 0.85^b^	2.03 ± 0.89^a,c^	1.27 ± 0.65^b^	**<0.01**
Limb dystonia	0.00 ± 0.00^b, c^	1.27 ± 1.31^a,c^	0.18 ± 0.40^b, c^	**<0.01**
Finger tapping	1.10 ± 0.30^b^	1.43 ± 0.50^a, c^	1.00 ± 0.00^b^	**<0.01**
Toe tapping	1.10 ± 0.30	1.23 ± 0.57	1.00 ± 0.00	0.19
Apraxia of hand movement	0.19 ± 0.40^b^	1.03 ± 0.76^a, c^	0.00 ± 0.00^b^	**<0.01**
Tremor in any part	0.23 ± 0.43	0.50 ± 0.57	0.27 ± 0.47	**0.04**
Neck rigidity or dystonia	1.77 ± 0.92^b^	1.23 ± 0.90^a^	1.64 ± 1.21	**0.02**
Arising from chair	2.10 ± 1.14^b^	1.27 ± 1.23^a^	1.64 ± 1.03	**<0.01**
Gait	1.87 ± 0.96	1.40 ± 0.81	1.91 ± 1.04	**0.04**
Postural instability	2.32 ± 1.19^b^	1.37 ± 1.07^a^	1.81 ± 1.25	**<0.01**
Sitting down	1.74 ± 1.03	1.17 ± 1.12	1.73 ± 1.01	**0.03**

Significantly different compared to ^a^PSP-RS, ^b^ CBS, ^c^ PSP-non-RS/CBS.

Demographic and clinical data were compared by *post-hoc* Bonferroni corrected ANOVA or Kruskal-Wallis test as appropriate. Chi-square analysis was used to check for significant differences in sex.

*CBS* corticobasal syndrome, *f* female, *m* male, *mo* months, MoCA Montreal Cognitive Assessment, *PSPRS progressive supranuclear palsy rating scale, PSP-RS* PSP Richardson’s syndrome, *SEADL* Schwab and England Activities of Daily Living Scale, *y* years

### 2.3 PET Imaging

#### 2.3.1 Radiosynthesis

Radiosynthesis of [^18^F]PI-2620 was achieved by nucleophilic substitution on a BOC-protected nitro precursor using an automated synthesis module (IBA, Synthera). The protecting group was cleaved under the radiolabelling conditions. The product was purified by semipreparative HPLC. Radiochemical purity was 99%. Non-decay corrected yields were about 35% with a molar activity of 8∙106 GBq/mmol at the end of synthesis.

#### 2.3.2 PET acquisition and preparation

The cohort of this study was scanned with a Biograph 64 or a mCT PET/CT scanner (both Siemens, Erlangen, Germany). A low-dose CT scan preceded the PET acquisition and served for attenuation correction. [^18^F]PI-2620 PET was performed in a full dynamic 0–60 min setting initiated upon intravenous injection (∼10 s) of 185 ± 10 MBq of the tracer in most of the patients and short imaging windows were used for a subset due to severe disability (11%). PET data were reconstructed iteratively (4 iterations, 21 subsets, 5.0 mm Gauss/ 5 iterations, 24 subsets, 5.0 mm Gauss) with a matrix size of 336 × 336 × 109/400 × 400 × 148, a voxel size of 1.018 × 1.018 × 2.027/1.018 × 1.018 × 1.500 mm^3^ / and a slice thickness of 2.027/ 1.500 mm. The previously evaluated single 20–40 min frame (Song et al., 2021) was used for all further analyses in order to allow inclusion of cases with short scans in severely disabled patients (20–40 min or 0–40 min scan).

#### 2.3.3 Image processing

All image data were processed and analysed using PMOD (version 3.9, PMOD Technologies Ltd., Zurich, Switzerland). For spatial normalisation, a tracer-specific 20–40 min template in the MNI space were created as described previously (Daerr et al., 2017). Based upon earlier experience, we created optimized templates by use of 35 randomly selected individuals with a structural high-resolution 3D MPRAGE. [^18^F]PI-2620 images were normalized to MNI space by applying a non-linear transformation (brain normalization settings: nonlinear warping, 8 mm input smoothing, equal modality, 16 iterations, frequency cutoff 3, regularization 1.0, no thresholding). The cerebellum (excluding the dentate nucleus and superior layers) was used as a reference region for scaling of [^18^F]PI-2620 images. Standardised uptake value ratios (SUVr) of all 246 supratentorial volumes of interest of the Brainnetome atlas were extracted. For further analysis regional SUVrs were converted into z-scores using tau-PET quantification of the healthy control cohort in order to ensure standardized region comparability. Initial judgement of tau-positivity was performed by a visual red of an experienced reader and supervision by an expert reader considering tau-PET signals in the frontal cortex, basal ganglia, substantia nigra and dentate nucleus. Basal ganglia positivity or positivity ≥ of the remaining regions defined the scan as positive.

### 2.4 Statistical analysis

Data were analysed using IBM SPSS Statistics for Windows (Version 28.0 Armonk, NY: IBM Corp.). Demographic as well as clinical data were compared between PSP-RS, CBS and PSP-non-RS/CBS patients by *post hoc* Bonferroni corrected ANOVA or Kruskal-Wallis test as appropriate. Chi-square analysis was used to check for significant differences in sex. Standard statistical significance level was set at *p* < 0.05.

To reduce the number of investigated cerebral regions and identify groups of similar brain regions with a confluent tau-PET signal based on regional SUVr z-scores, our set of 246 supratentorial cerebral regions was subjected to a principal component analysis (PCA) with direct oblimin rotation. Following the advice of Stevens (Stevens, 2009) factor loadings above 0.4 were regarded significant. Variables with factor loadings below 0.4 were eliminated from the analysis and the PCA run anew. Components were labelled *post hoc* according to the distribution of cerebral regions. Individual regression-based component scores were calculated (Thurstone, 1934). To visualize the similarity of variables assigned to a specific component during PCA, multidimensional scaling (MDS) using Euclidean distances was performed.

False discovery rate-corrected Spearman’s tests (q < 0.05) were used to explore significant correlations between the calculated individual component scores and the items of the PSPRS in the groups of PSP-RS and CBS patients as well as the whole study sample. Due to the comparatively low sample size and the mixture of clinical phenotypes, no correlation analysis was performed for the subgroup of PSP-non-RS/CBS patients.

### 2.5 Data availability

The data that support the findings of this study are available from the corresponding author upon reasonable request and submission of a formal project outline. Data are not publicly available due to privacy restrictions.

## 3 Results

Clinical scorings of the study population are provided in Table 1. Groups did not differ in terms of age, sex, years of education, disease duration, MoCA and SEADL. PSP-RS patients had higher scores of the PSPRS items ocular voluntary upward, downward, and left and right command movement compared to PSP-non-RS/CBS and CBS patients. Furthermore, they had higher total PSPRS scores as well as higher scores on the items dysphagia for solids, falls, dysphagia, eyelid dysfunction, neck rigidity or dystonia, arising from chair and postural instability compared to CBS patients. In contrast, CBS patients scored significantly higher on the items limb rigidity, limb dystonia, finger tapping, and apraxia of hand movement compared to the other patient groups. PSP-non-RS/CBS patients had higher scores of the item limb dystonia compared to PSP-RS patients. Kruskal-Wallis test detected significant group differences of the items withdrawal, tremor in any part, gait and sitting down; however, *post-hoc* Mann-Whitney tests did not show significant pairwise differences. All but 8 patients (11.1%, 4 PSP-RS patients and 4 CBS patients) were tau positive on visual inspection. Data on β-amyloid status were available in 16/31 PSP-RS patients. In 14 patients a primary AD pathology could be excluded by evaluation of β-amyloid in cerebrospinal fluid or [^18^F]flutemetamol and [^18^F]florbetaben PET respectively. Two patients showed a reduced Aβ42/40 ratio.

### 3.1 Principal component analysis and multidimensional scaling

**Fig. 1:**
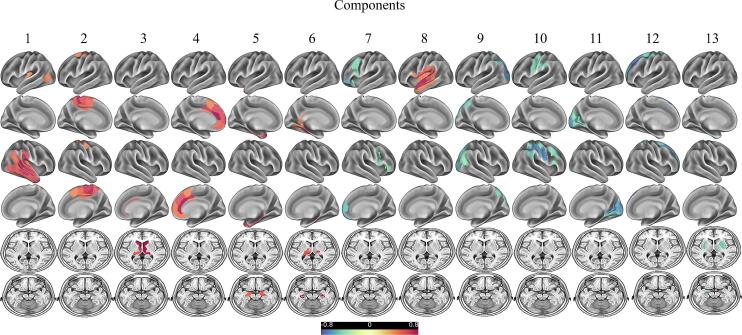
Principal components of [^18^F]-PI-2620 SUVr z-scores. Rotated regional weights of [^18^F]PI-2620 SUVr z-score components identified by applying principal component analysis on 246 cerebral regions. The colours represent the region-specific weights (range from −0.8 to 0.8) on each component.

PCA with direct oblimin rotation applied to the tau-PET data revealed the presence of 26 components with eigenvalues above 1.0. However, 13 of these components contained<3 variables and were consecutively excluded from the analysis, leaving a 13-component solution which explained 85.2% of variance. 64 cerebral regions were excluded as their factor loadings were below 0.4. A detailed description of the different components is provided in Fig. 1 and Supplementary Table 1.

**Fig. 2:**
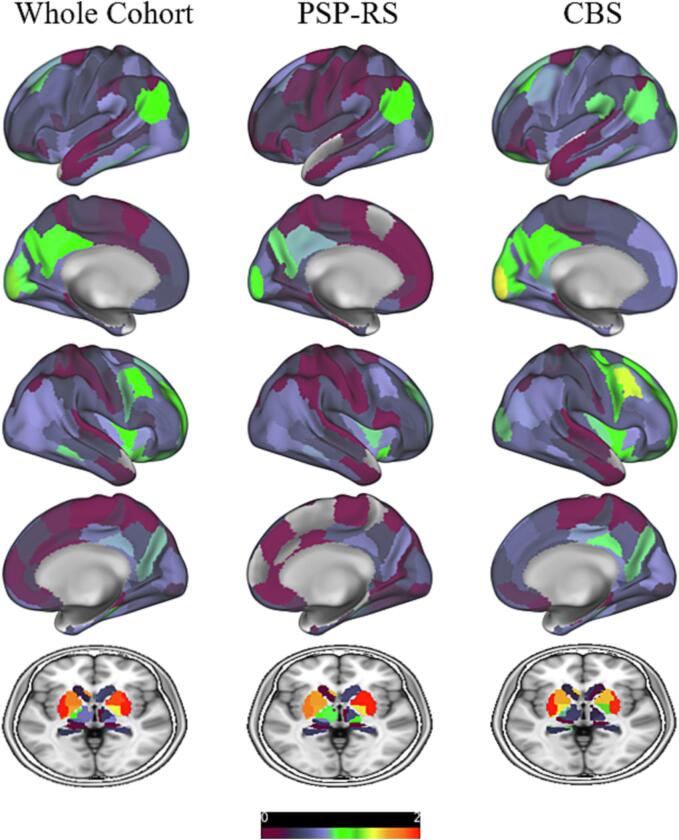
Regional SUVr Z-score maps of [^18^F]-PI-2620 of the whole cohort as well as the group of PSP-RS and CBS patients.

**Fig. 3:**
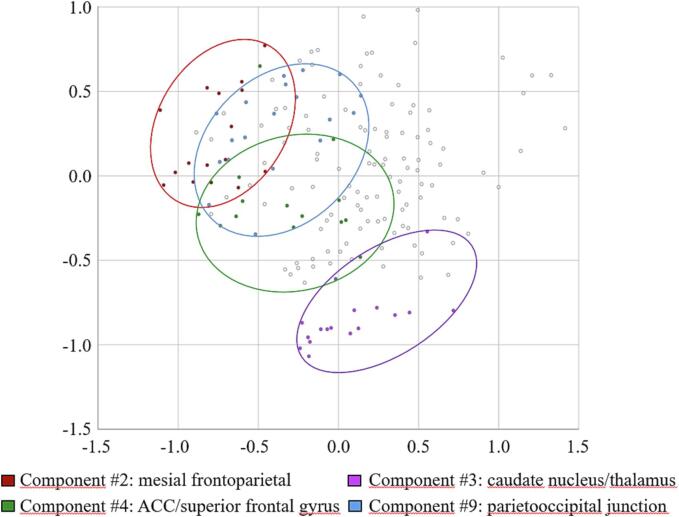
Multidimensional scaling of [^18^F]PI-2620 SUVr z-scores. Two-dimensional spatial representation based on the similarity of [^18^F]PI-2620 SUVr z-scores as revealed by MDS. SUVr z-scores that have been assigned to a specific component by PCA are colour-coded. For the sake of clarity, only the four components correlating with clinical symptoms in the whole cohort are depicted.

In summary, the resulting components comprised the following regions: component #1 = right lateral temporal lobe (inferior/middle and superior temporal gyrus) (variance explained 41.1%), component #2 = mesial frontoparietal lobes (variance explained 16.9%), component #3 = caudate nucleus/thalamus (variance explained 6.5%), component #4 = medial superior frontal gyrus and adjacent anterior cingulate cortex/orbital gyrus (variance explained 3.9%), component #5 = parahippocampal gyrus (variance explained 3.3%), component #6 = hippocampus/thalamus (variance explained 2.9%), component #7 = inferolateral frontal lobe (variance explained 2.1%), component #8 = left parietotemporal junction (variance explained 1.8%), component #9 = parietooccipital junction (variance explained 1.6%), component #10 = lateral frontoparietal lobes (variance explained 1.5%), component #11 = medio ventral occipital cortex (variance explained 1.4%), component #12 = superior and middle frontal gyrus (variance explained 1.3%), component #13 = basal ganglia (variance explained 1.1%). Regions of confluent tau-PET signal as identified by PCA do not necessarily reflect the regions of highest tau load compared to the control group. For comparison, Fig. 2 shows the z-score maps of PSP-RS and CBS patients as well as the whole cohort indicating respective effect sizes. MDS confirmed the grouping of cerebral regions as reasonable (normalized raw stress 0.061) (Fig. 3). Separate PCAs of the subgroup of PSP-RS and CBS patients respectively resulted in similar groupings of variables in the individual components (Supplementary Table 2 and 3).

### 3.2 Correlation analysis

**Fig. 4:**
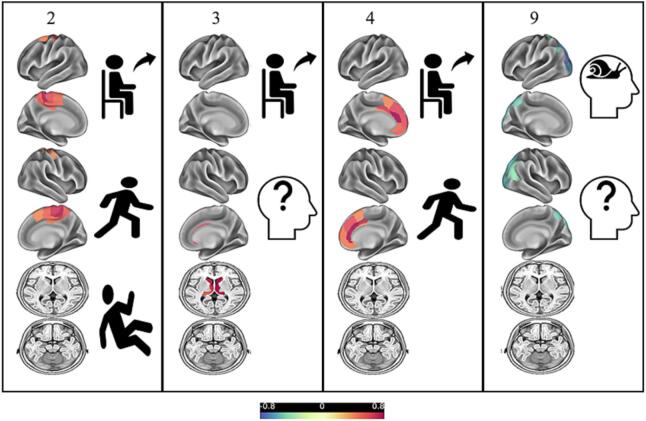
Schematic illustration of the correlation between regional tau-tracer uptake and clinical signs. Depicted are the components #2 (mesial frontoparietal lobes), #3 (caudate nucleus/thalamus), #4 (medial superior frontal gyrus and adjacent anterior cingulate cortex/orbital gyrus) and #9 (parietotemporal junction) correlating with the signs arising from chair (), gait (), postural instability (), disorientation () and bradyphrenia ().

Correlation analyses performed within the whole study sample showed a positive correlation of the signs arising from chair and gait respectively with component #2 (mesial frontoparietal lobes; *r_s_* = 0.299, *p* = 0.011 and *r_s_* = 0.310, *p* = 0.008) and #4 (medial superior frontal gyrus and adjacent anterior cingulate cortex/orbital gyrus; *r_s_* = 0.427, *p* < 0.001 and *r_s_* = 0.407, *p* < 0.001). Furthermore, postural instability was positively correlated with component #2 (mesial frontoparietal lobes; *r_s_* = 0.383, *p* = 0.001). While the signs disorientation and bradyphrenia showed a positive correlation with component #9 (parietooccipital junction; *r_s_* = 0.313, *p* = 0.007 and *r_s_* = 0.367, *p =* 0.002), disorientation and arising from chair were negatively correlated with component #3 (caudate nucleus/thalamus; *r_s_* = -0.340, *p* = 0.004 and *r_s_* = -0.312, *p* = 0.008). A schematic illustration of the correlation between regional tau-tracer uptake and clinical signs is given in Fig. 4.

**Fig. 5:**
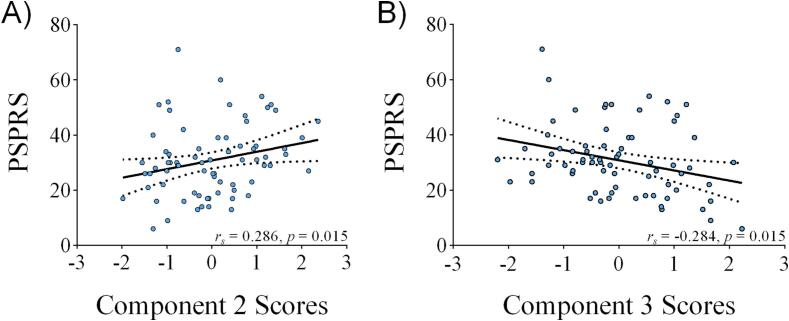
Correlation of clinical ratings by PSPRS with component scores. Total PSPRS showed a trend towards a positive correlation with component #2 (mesial frontoparietal lobes; A) and a trend towards a negative correlation with component #3 (caudate nucleus/thalamus; B) but did not survive the significance threshold for multiple comparisons.

Total PSPRS showed a trend towards a positive correlation with component #2 (mesial frontoparietal lobes) and a trend towards a negative correlation with component #3 (caudate nucleus/thalamus) but did not survive the significance threshold for multiple comparisons (Fig. 5).

Furthermore, age was positively correlated with component #1 (right lateral temporal lobe; *r_s_* = 0.354, *p* = 0.002) and #13 (basal ganglia; *r_s_* = 0.400, *p* < 0.001) and negatively correlated with component #3 (caudate nucleus/thalamus; *r_s_* = -0.446, *p* < 0.001). The MoCA showed a positive correlation with component #3 (caudate nucleus/thalamus; *r_s_* = 0.411, *p* = 0.001) while the SEADL showed a negative correlation with component #2 (mesial frontoparietal lobes; *r_s_* = -0.354, *p* = 0.001) and a positive correlation with component #3 (caudate nucleus/thalamus; *r_s_* = 0.342, *p* = 0.004). No significant correlation with the calculated component scores could be detected for disease duration and the duration of education.

In PSP-RS patients a negative correlation of the sign disorientation with component #7 (inferolateral frontal lobe; *r_s_* = -0.594, *p* < 0.001), could be detected. Furthermore, apraxia of hand movement was negatively correlated with component #3 (caudate nucleus/thalamus; *r_s_* = -0.511, *p* = 0.003) and #8 (left parietotemporal junction; *r_s_* = -0.502, *p* = 0.004). Arising from chair showed a positive correlation with component #1 (right lateral temporal lobe; *r_s_* = 0.523, *p_s_* = 0.003) while gait showed a positive correlation with component 4 (medial superior frontal gyrus and adjacent anterior cingulate cortex/orbital gyrus; *r_s_* = 0.639, *p* < 0.001).

In CBS patients a negative correlation of component #3 (caudate nucleus/thalamus) with the signs arising from chair (*r_s_* = -0.654, *p* < 0.001), gait (*r_s_ =* -0.606, *p* < 0.001), postural instability (*r_s_* = -0.547, *p* = 0.002) and sitting down (*r_s_* = -0.547, *p* = 0.001)*,* as well as with total PSPRS (*r_s_* = -0.700, *p* < 0.001) could be detected. Arising from chair was also positively correlated with component #4 (medial superior frontal gyrus and adjacent anterior cingulate cortex/orbital gyrus; *r_s_* = 0.480, *p* = 0.007).

## 4 Discussion

We present the first study in 4R-tauopathies demonstrating a correlation of clinical symptomatology with [^18^F]PI-2620 binding in specific brain regions, defined by a data-driven selection.

Like in previous studies (Brendel et al., 2020, Palleis et al., 2021) total PSPRS was not significantly correlated with [^18^F]PI-2620 binding when correcting for multiple comparisons. However, there was a trend towards a positive correlation with tracer uptake in mesial frontoparietal lobes and towards a negative correlation with tracer uptake in caudate nucleus and thalamus. Tracer uptake in mesial frontoparietal lobes was specifically associated with worse performances in arising from chair, gait, and postural instability. The mesial frontoparietal lobe consists of the primary motor, premotor and supplementary motor cortex, regions that are part of the supraspinal locomotor network (Bohnen and Jahn, 2013), as well as the post- and paracentral cortex. These regions play a key role in movement planning and spatial navigation and have been shown to be affected by tau pathology in PSP (Kovacs et al., 2020) and CBD (Kouri et al., 2011). Moreover, consistent with our findings, a previous study in PSP patients demonstrated flortaucipir uptake in these regions to be correlated with worse gait performances as well as greater postural instability (Sintini et al., 2021).

Worse performances in arising from chair and gait were furthermore associated with tracer uptake in bilateral anterior cingulate cortex and medial superior frontal cortex. The anterior cingulate cortex has widespread connections to the prefrontal, premotor and supplementary motor cortex, parietal lobe, amygdala and hypothalamus (Margulies et al., 2007, Jin et al., 2018) and plays an important role in a variety of movement-related functions like attention, executive function, action selection and initiation and performance monitoring (Tian et al., 2017, Srinivasan et al., 2013). Also, the medial superior frontal cortex is involved in spatial processing, attention as well as executive function (du Boisgueheneuc et al., 2006, Nagahama et al., 1999) and is therefore relevant for planning and execution of movements. In line with our data, previous neuroimaging studies have revealed MRI volumes, hypometabolism and flortaucipir uptake of the medial superior frontal cortex including the anterior cingulate cortex to contribute to the variance in stride length and difficulties in gait initiation in PSP (Sintini et al., 2021, Palmisano et al., 2020).

While bradyphrenia is typically brought into connection with striatofrontal dysfunction (Dubois et al., 1988), in our study bradyphrenia and disorientation correlated with tracer uptake near the parietooccipital junction, consisting of the inferior and superior parietal lobule including the precuneus and the lateral occipital cortex. Recent studies have revealed cortical activation related to orientation in space, time and person in the precuneus, regions within the inferior parietal lobe as well as the occipital lobe, with a posterior-anterior axis of activation for space, person and time (Peer et al., 2015). The precuneus and inferior parietal lobule furthermore represent nodes of the default mode network which is involved in self-referential processes (Buckner and DiNicola, 2019). A disruption of these closely related networks may result in both bradyphrenia and disorientation.

While counter-intuitive at first glance, tracer uptake in the caudate nucleus and thalamus was inversely correlated with the signs arising from chair and disorientation in our cohort. Our findings are however in agreement with a previous study showing flortaucipir uptake in the caudate nucleus to be positively correlated with velocity in PSP patients (Sintini et al., 2021). Progressive atrophy of basal ganglia structures and the thalamus is a frequent finding in PSP and CBD (Dutt et al., 2016). Therefore, the negative correlation of tracer uptake with symptomatology may be due to an increasing atrophy in these regions leading a) to a secondary process that is counteracting the increase of tau deposits and to b) partial volume effects. This may explain a relative decrease of tracer uptake in the disease course.

The evaluation of subgroups, i.e. PSP-RS and CBS, revealed specific correlations depending on the respective phenotype. While in patients with CBS, the main finding was a negative association of tracer binding in the component consisting of caudate nucleus and thalamus and a positive association of tracer binding in the component consisting of the medial frontal cortex with gait and motor signs, various correlations of clinical signs with tracer binding in specific cerebral regions could be detected in patients with PSP-RS. These findings suggest that a profound knowledge of the clinical phenotype is essential for the interpretation, especially of longitudinal [^18^F]PI-2620 PET findings. Therefore, a close cooperation of neurologists and nuclear medicine physicians is necessary for optimal diagnosis and treatment decisions in patients with a 4R-tauopathy.

To be of use for the evaluation of therapeutic agents in future clinical trials, a biomarker should aid the early detection of neurodegenerative processes, the early differentiation of the underlying pathology, and should correlate with clinical disease progression (van Eimeren et al., 2019). Previous correlative results between tau tracer binding and clinical severity in 4R-tauopathies have been inconsistent (Palleis et al., 2021, Brendel et al., 2017, Schönecker et al., 2019, Schonhaut et al., 2017). However, to date only composite scores like the PSPRS were investigated. While composite scores may be useful to assess global disease severity in patients, a correlation with imaging findings is not to be expected as differing anatomical distributions of pathological brain changes underlie the individual signs and symptoms (Weintraub and Mesulam, 2009). The current study, however, provides evidence of specific clinical signs, especially motor signs and symptoms to be associated with [^18^F]PI-2620 tracer uptake in specific brain regions. This holds true for the cohort as a whole, as well as for the subgroup of patients with PSP-RS and CBS and underlines the potential of [^18^F]PI-2620 PET as a biomarker assessing phenotype and disease stage and progression in the most common 4R-tauopathies.

The temporal lobe resulting as the first component in PCA was quite surprising. However, the fact that the following component represent typical regions affected in 4R-tauopathies reassured us about the correctness of the analysis. Components as identified by PCA do not necessarily reflect regions of highest tau load compared to controls. While the temporal lobe is not the primary region of tau deposits, these may be detected especially in more advances disease stages or in a subset of patients with specific phenotypes like PSP-F (Kovacs et al., 2020). This heterogeneity seems to have led to a high variance regarding tau deposition in the temporal lobe and thereby to the temporal lobe resulting as the first component of PCA. Forthermore, while AD pathology has been excluded in the majority of patients a beginning AD-copathology or primary age related tauopathy in a subset of patients may have additionally contributed to a high variance in tracer uptake in temporal regions.

A limitation of the current study that needs to be considered is the lack of histopathological validation which reflects the moderate clinical severity within our patients, most of whom are alive at the time being. However, the lack of neuropathological confirmation of diagnosis is a common problem in studies investigating rare neurodegenerative disorders, in particular when innovative PET tracers are investigated as in this study. There remains the possibility that some cases had a mismatch of clinical diagnosis and underlying pathology. However, a tight clinical follow-up after initial diagnosis ensured us about the validity of clinical diagnosis (duration 24.1 ± 20.7 moths, frequency every 4.6 ± 3.1 months).

Off-target binding, especially to monoamine-oxidase B in basal ganglia structures, has been a major limitation associated with first-generation tau tracers. Recent studies, however, indicate that monoamine-oxidase B off-target binding has been overcome in next generation tau-tracers like [^18^F]PI-2620 (Yap et al., 2021, Kroth et al., 2019). Moreover, cortical and subcortical tracer binding has been shown to be low to absent in healthy controls (Brendel et al., 2020). Nonetheless, binding to other potential off-target sources like neuromelanin, iron or microhemorrhage cannot be excluded and may have influenced the analyses performed. Another limitation is the lack of standardized volumetric MRI data to confirm caudate and thalamic atrophy. However, progressive atrophy of midbrain, basal ganglia structures and thalamus is frequent in 4R-tauopathies (Stamelou et al., 2021) and is therefore also likely in our cases. Due to the lack of standardized MRI data, it was also not possible to apply partial volume effect correction (Rullmann et al., 2016) to the PET data. Although partial volume effect correction could likely alter the observed negative associations between regional tau-PET signals and clinical scores, this technique is not available in most clinical settings. Thus, we decided to focus on tau-PET data without adjustment for atrophy.

Despite these limitations, our data reveal that data driven [^18^F]PI-2620 tau-PET topology correlates with symptomatology in 4R-tauopathies. As the cerebral regions correlating with symptomatology differ depending on the clinical phenotype, a precise knowledge of signs and symptoms is necessary when interpreting [^18^F]PI-2620 PET results and renders a close cooperation of the treating neurology and nuclear medicine specialists necessary. Longitudinal studies will be needed to address whether a deterioration of signs and symptoms over time can be monitored by [^18^F]PI-2620 PET in 4R-tauopathies on an individual basis.

## Funding

The Lüneburg Heritage and Friedrich-Baur-Stiftung have supported the work of C.P. The Lüneburg Heritage and the Ehrmann Foundation have supported the work of S.K. This work was funded by the Deutsche Forschungsgemeinschaft (DFG, German Research Foundation) under Germany’s Excellence Strategy within the framework of the Munich Cluster for Systems Neurology (EXC 2145 SyNergy – ID 390857198).

## CRediT authorship contribution statement

**Sonja Schönecker:** Conceptualization, Formal analysis, Writing – original draft. **Carla Palleis:** Resources, Writing – review & editing. **Nicolai Franzmeier:** Writing – review & editing, Visualization. **Sabrina Katzdobler:** Resources, Writing – review & editing. **Christian Ferschmann:** Resources, Writing – review & editing. **Sebastian Schuster:** Resources, Writing – review & editing. **Anika Finze:** Resources, Writing – review & editing. **Maximilian Scheifele:** Resources, Writing – review & editing. **Catharina Prix:** Resources, Writing – review & editing. **Urban Fietzek:** Resources, Writing – review & editing. **Endy Weidinger:** Resources, Writing – review & editing. **Georg Nübling:** Resources, Writing – review & editing. **Jonathan Vöglein:** Resources, Writing – review & editing. **Marianne Patt:** Methodology, Writing – review & editing. **Henryk Barthel:** Methodology, Writing – review & editing. **Osama Sabri:** Methodology, Writing – review & editing. **Adrian Danek:** Resources, Writing – review & editing. **Günter U. Höglinger:** Resources, Writing – review & editing. **Matthias Brendel:** Conceptualization, Methodology, Writing – review & editing, Funding acquisition. **Johannes Levin:** Resources, Writing – review & editing, Funding acquisition, Supervision, Project administration.

## Declaration of Competing Interest

Johannes Levin reports speaker fees from Bayer Vital, Biogen and Roche, consulting fees from Axon Neuroscience and Biogen, author fees from Thieme medical publishers and W. Kohlhammer GmbH medical publishers, non-financial support from Abbvie and compensation for duty as part-time CMO from MODAG and being beneficiary of the phantom share program of MODAG GmbH, outside the submitted work. Matthias Brendel received speaker honoraria from Roche, GE healthcare and Life Molecular Imaging and is an advisor of Life Molecular Imaging. Sabrina Katzdobler has received travel support from Life Molecular Imaging. Osama Sabri receives research support from Life Molecular Imaging. Henryk Barthel received speaker honoraria from Novartis/AAA and reader honoraria from Life Molecular Imaging. Nicolai Franzmeier received research funding from Avid Radiopharmaceuticals and consulting fees from Merck.

## Data Availability

Data will be made available on request.
