# Supplementary material for: Symptomatology in 4-repeat tauopathies is associated with data-driven topology of [18F]-PI-2620 tau-PET signal
Source: Neuroimage Clin. 2023 Apr 11;38:103402. doi: 10.1016/j.nicl.2023.103402 (PMC10300609; doi:10.1016/j.nicl.2023.103402)
Supplement: Supplementary data 1 [file mmc1.docx]

Supplementary Material

| **Tab. 1 Regional components defined by PCA** | | | | | | |
| --- | --- | --- | --- | --- | --- | --- |
| **Regioncode** | **Factor loadings** | **X** | **Y** | **Z** | **Region** | **Subregion** |
| **Component #1: Right lateral temporal lobe** | | | | | | |
| cpSTS_r | 0.721 | 148 | 87 | 98 | Posterior Superior Temporal Sulcus | caudoposterior superior temporal sulcus |
| rpSTS_r | 0.706 | 143 | 91 | 106 | Posterior Superior Temporal Sulcus | rostroposterior superior temporal sulcus |
| A21c_r | 0.700 | 155 | 99 | 123 | Middle Temporal Gyrus | caudal area 21 |
| aSTS_r | 0.649 | 149 | 112 | 119 | Middle Temporal Gyrus | anterior superior temporal sulcus |
| A40rv_r | 0.642 | 146 | 101 | 84 | Inferior Parietal Lobule | rostroventral area 40 |
| A20r_r | 0.615 | 131 | 129 | 152 | Inferior Temporal Gyrus | rostral area 20 |
| A22c_r | 0.614 | 157 | 107 | 103 | Superior Temporal Gyrus | caudal area 22 |
| A20cv_r | 0.606 | 145 | 97 | 135 | Inferior Temporal Gyrus | caudoventral of area 20 |
| A20cl_r | 0.602 | 151 | 88 | 127 | Inferior Temporal Gyrus | caudolateral of area 20 |
| V5_MT_plus_r | 0.601 | 139 | 58 | 111 | Lateral Occipital Cortex | area V5/MT+ |
| A41_42_r | 0.587 | 145 | 104 | 99 | Superior Temporal Gyrus | area 41/42 |
| A21r_r | 0.582 | 142 | 134 | 141 | Middle Temporal Gyrus | rostral area 21 |
| A37vl_r | 0.559 | 145 | 71 | 118 | Inferior Temporal Gyrus | ventrolateral area 37 |
| A37dl_r | 0.558 | 151 | 74 | 107 | Middle Temporal Gyrus | dorsolateral area 37 |
| A22r_r | 0.556 | 147 | 116 | 114 | Superior Temporal Gyrus | rostral area 22 |
| A20il_r | 0.553 | 145 | 117 | 141 | Inferior Temporal Gyrus | intermediate lateral area 20 |
| A37lv_r | 0.537 | 133 | 79 | 129 | Fusiform Gyrus | ventrolateral area 37 |
| G_r | 0.508 | 128 | 110 | 101 | Insular Gyrus | hypergranular insular |
| A20iv_r | 0.495 | 137 | 115 | 142 | Temporal Lobe | intermediate ventral area 20 |
| A39rv_r | 0.495 | 144 | 73 | 86 | Inferior Parietal Lobule | rostroventral area 39 |
| V5_MT_plus_l | 0.469 | 45 | 54 | 107 | Lateral Occipital Cortex | area V5/MT+ |
| G_l | 0.405 | 55 | 108 | 99 | Insular Gyrus | hypergranular insular |
| **Component #2: Mesial frontoparietal lobes** | | | | | | |
| A1_2_3ll_r | 0.727 | 101 | 92 | 55 | Paracentral Lobule | area 1/2/3 |
| A1_2_3ll_l | 0.725 | 84 | 89 | 51 | Paracentral Lobule | area 1/2/3 |
| A4ll_r | 0.691 | 96 | 105 | 48 | Paracentral Lobule | area 4 |
| A4ll_l | 0.619 | 87 | 104 | 48 | Paracentral Lobule | area 4 |
| A4t_r | 0.599 | 106 | 105 | 38 | Precentral Gyrus | area 4 |
| A5m_l | 0.557 | 83 | 79 | 52 | Precuneus | medial area 5 |
| A5m_r | 0.556 | 99 | 80 | 51 | Precuneus | medial area 5 |
| A4ul_l | 0.555 | 65 | 102 | 46 | Precentral Gyrus | Area 4 |
| A23c_l | 0.545 | 84 | 104 | 68 | Cingulate Gyrus | caudal area 23 |
| A6m_r | 0.532 | 99 | 123 | 49 | Superior Frontal Gyrus | medial area 6 |
| A6m_l | 0.523 | 85 | 122 | 51 | Superior Frontal Gyrus | medial area 6 |
| A23c_r | 0.499 | 97 | 107 | 69 | Cingulate Gyrus | caudal area 23 |
| A6dl_l | 0.451 | 73 | 126 | 43 | Superior Frontal Gyrus | dorsolateral area 6 |
| A8m_r | 0.431 | 99 | 143 | 54 | Superior Frontal Gyrus | medial area 8 |
| A1_2_3tru_r | 0.423 | 111 | 94 | 40 | Postcentral Gyrus | area 1/2/3 |
| A4ul_r | 0.409 | 125 | 107 | 51 | Precentral Gyrus | area 4 |
| **Component #3: Caudate nucleus/thalamus** | | | | | | |
| dCa_r | 0.986 | 105 | 133 | 94 | Basal Ganglia | dorsal caudate |
| cTtha_r | 0.985 | 101 | 113 | 95 | Thalamus | caudal temporal thalamus |
| cTtha_l | 0.955 | 80 | 106 | 96 | Thalamus | Caudal temporal thalamus |
| dCA_l | 0.928 | 77 | 130 | 92 | Basal Ganglia | dorsal caudate |
| rTtha_l | 0.870 | 84 | 114 | 102 | Thalamus | rostral temporal thalamus |
| Otha_r | 0.869 | 104 | 101 | 102 | Thalamus | occipital thalamus |
| rTtha_r | 0.868 | 94 | 115 | 103 | Thalamus | rostral temporal thalamus |
| mPFtha_r | 0.811 | 98 | 117 | 103 | Thalamus | media pre-frontal thalamus |
| lPFtha_r | 0.785 | 103 | 111 | 102 | Thalamus | lateral pre-frontal thalamus |
| PPtha_r | 0.734 | 106 | 103 | 103 | Thalamus | posterior parietal thalamus |
| vCa_l | 0.729 | 79 | 142 | 108 | Basal Ganglia | ventral caudate |
| vCa_r | 0.674 | 106 | 142 | 111 | Basal Ganglia | ventral caudate |
| mPFtha_l | 0.651 | 84 | 116 | 103 | Thalamus | medial pre-frontal thalamus |
| A24rv_r | 0.634 | 96 | 149 | 96 | Cingulate Gyrus | rostroventral area 24 |
| PPtha_l | 0.636 | 75 | 104 | 103 | Thalamus | posterior parietal thalamus |
| Otha_l | 0.464 | 76 | 100 | 105 | Thalamus | occipital thalamus |
| NAC_r | 0.449 | 105 | 136 | 117 | Basal Ganglia | nucleus accumbens |
| **Component #4: Medial superior frontal gyrus/anterior cingulate cortex/orbital gyrus** | | | | | | |
| A32p_l | 0.800 | 85 | 162 | 87 | Cingulate Gyrus | pregenual area 32 |
| A32sg_r | 0.779 | 96 | 169 | 101 | Cingulate Gyrus | subgenual area 32 |
| A32p_r | 0.772 | 96 | 155 | 80 | Cingulate Gyrus | pregenual area 32 |
| A24cd_r | 0.700 | 95 | 133 | 70 | Cingulate Gyrus | caudodorsal area 24 |
| A24cd_l | 0.642 | 86 | 134 | 71 | Cingulate Gyrus | caudodorsal area 24 |
| A9m_l | 0.634 | 87 | 163 | 69 | Superior Frontal Gyrus | medial area 9 |
| A10m_l | 0.622 | 84 | 183 | 93 | Superior Frontal Gyrus | medial area 10 |
| A9m_r | 0.614 | 97 | 165 | 73 | Superior Frontal Gyrus | medial area 9 |
| A14m_l | 0.560 | 84 | 182 | 114 | Orbital Gyrus | medial area 14 |
| A32sg_l | 0.506 | 87 | 167 | 110 | Cingulate Gyrus | subgenual area 32 |
| A10m_r | 0.478 | 99 | 186 | 94 | Superior Frontal Gyrus | medial area 10 |
| A14m_r | 0.472 | 97 | 176 | 114 | Orbital Gyrus | medial area 14 |
| A24rv_l | 0.443 | 88 | 136 | 84 | Cingulate Gyrus | rostroventral area 24 |
| A8m_l | 0.438 | 87 | 142 | 54 | Superior Frontal Gyrus | medial area 8 |
| A8m_r | 0.426 | 99 | 143 | 54 | Superior Frontal Gyrus | medial area 8 |
| A24rv_r | 0.407 | 96 | 149 | 96 | Cingulate Gyrus | rostroventral area 24 |
| **Component #5: Parahippocampal gyrus** | | | | | | |
| A28_34_r | 0.896 | 109 | 118 | 138 | Parahippocampal Gyrus | area 28/34 |
| A28_34_l | 0.885 | 72 | 117 | 139 | Parahippocampal Gyrus | area 28/34 |
| TI_r | 0.730 | 112 | 130 | 145 | Parahippocampal Gyrus | temporal agranular insular cortex |
| TI_l | 0.714 | 68 | 131 | 140 | Parahippocampal Gyrus | temporal agranular insular cortex |
| A35_36c_r | 0.704 | 117 | 105 | 136 | Parahippocampal Gyurs | caudal area 35/36 |
| TH_r | 0.683 | 109 | 92 | 121 | Parahippocampal Gyrus | medial posterior parahippocampal cortex |
| A38m_r | 0.677 | 122 | 144 | 142 | Superior Temporal Gyrus | medial area 38 |
| A38m_l | 0.677 | 59 | 143 | 142 | Superior Temporal Gyrus | medial area 38 |
| rHipp_r | 0.546 | 113 | 116 | 129 | Hippocampus | rostral hippocampus |
| A38l_l | 0.526 | 47 | 139 | 127 | Superior Temporal Gyrus | lateral area 38 |
| A35_36r_r | 0.526 | 118 | 120 | 142 | Parahippocampal Gyrus | rostral area 35/36 |
| mAmyg_r | 0.506 | 110 | 126 | 128 | Amygdala | medial amygdala |
| mAmyg_l | 0.589 | 72 | 126 | 128 | Amygdala | medial amygdala |
| rHipp_l | 0.479 | 69 | 114 | 127 | Hippocampus | rostral hippocampus |
| A35_36r_l | 0.472 | 64 | 122 | 142 | Parahippocampal Gyrus | rostral area 35/36 |
| A35_36c_l | 0.432 | 66 | 104 | 135 | Parahippocampal Gyrus | caudal area 35/36 |
| **Component #6: Hippocampus/thalamus** | | | | | | |
| TL_l | 0.804 | 63 | 97 | 127 | Parahippocampal Gyrus | lateral posterior parahippocampal gyrus |
| cHipp_l | 0.800 | 63 | 98 | 119 | Hippocampus | caudal hippocampus |
| cHipp_r | 0.734 | 120 | 101 | 120 | Hippocampus | caudal hippocampus |
| Stha_r | 0.661 | 109 | 106 | 106 | Thalamus | sensory thalamus |
| Otha_l | 0.651 | 76 | 100 | 105 | Thalamus | occipital thalamus |
| TH_L | 0.611 | 74 | 89 | 119 | Parahippocampal Gyrus | medial posterior parahippocampal cortex |
| Stha_l | 0.607 | 73 | 105 | 105 | Thalamus | sensory thalamus |
| TL_r | 0.549 | 120 | 98 | 127 | Parahippocampal Gyrus | lateral posterior parahippocampal cortex |
| PPtha_l | 0.518 | 75 | 104 | 103 | Thalamus | posterior parietal thalamus |
| A35_36c_l | 0.496 | 66 | 104 | 135 | Parahippocampal Gyrus | caudal area 35/36 |
| rLinG_l | 0.468 | 74 | 68 | 116 | Medio Ventral Occipital Cortex | rostral lingual gyrus |
| IPFtha_l | 0.459 | 80 | 113 | 106 | Thalamus | lateral pre-frontal thalamus |
| A23v_l | 0.410 | 83 | 80 | 100 | Cingulate Gyrus | ventral area 23 |
| **Component #7: Inferolateral frontal lobe** | | | | | | |
| A11m_r | -0.612 | 98 | 185 | 124 | Orbital Gyrus | medial area 11 |
| A45r_l | -0.592 | 42 | 164 | 110 | Inferior Frontal Gyrus | rostral area 45 |
| A10m_r | -0.558 | 99 | 186 | 94 | Superior Frontal Gyrus | medial area 10 |
| A12_47l_l | -0.536 | 50 | 160 | 117 | Orbital Gyrus | lateral area 12/47 |
| A44v_r | -0.499 | 145 | 141 | 98 | Inferior Frontal Gyrus | ventral area 44 |
| IFJ_l | -0.494 | 50 | 141 | 72 | Middle Frontal Gyrus | inferior frontal junction |
| A44v_l | -0.468 | 39 | 141 | 101 | Inferior Frontal Gyrus | ventral area 44 |
| A45r_r | -0.455 | 142 | 164 | 108 | Inferior Frontal Gyrus | rostral area 45 |
| A6cvl_r | -0.441 | 142 | 134 | 78 | Precentral Gyrus | caudal ventrolateral area 6 |
| A44d_l | -0.418 | 45 | 141 | 84 | Inferior Frontal Gyrus | dorsal area 44 |
| A44op_r | -0.408 | 133 | 150 | 105 | Inferior Frontal Gyrus | opercular area 44 |
| **Component #8: Left parietotemporal junction** | | | | | | |
| TE1.0_TE1.2_l | 0.825 | 41 | 117 | 107 | Superior Temporal Gyrus | TE1.0 and TE1.2 |
| A22r_l | 0.752 | 36 | 125 | 118 | Superior Temporal Gyrus | rostral area 22 |
| A41_42_l | 0.651 | 37 | 96 | 96 | Superior Temporal Gyrus | area 41/42 |
| A4tl_l | 0.612 | 39 | 128 | 100 | Precentral Gyrus | area 4 |
| A1_2_3tonIa_l | 0.600 | 35 | 114 | 92 | Postcentral Gyrus | area 1/2/3 |
| A22c_l | 0.571 | 29 | 95 | 101 | Superior Temporal Gyrus | caudal area 22 |
| aSTS_l | 0.548 | 32 | 109 | 118 | Middle Temporal Gyrus | anterior superior temporal sulcus |
| A40rv_l | 0.507 | 37 | 97 | 86 | Inferior Parietal Lobule | rostroventral area 40 |
| Dlg_l | 0.469 | 53 | 120 | 100 | Insular Gyrus | dorsal granular insular |
| A38l_l | 0.469 | 35 | 139 | 127 | Superior Temporal Gyrus | lateral area 38 |
| A21c_l | 0.463 | 26 | 98 | 120 | Middle Temporal Gyrus | caudal area 21 |
| A44v_l | 0.450 | 39 | 141 | 101 | Inferior Frontal Gyrus | ventral area 44 |
| G_l | 0.436 | 55 | 108 | 99 | Insular Gyrus | hypergranular insular |
| A21r_l | 0.421 | 38 | 131 | 137 | Middle Temporal Gyrus | rostral area 21 |
| rpSTS_l | 0.418 | 37 | 88 | 105 | Posterior Superior Temporal Sulcus | rostroposterior superior temporal sulcus |
| **Component #9: Parietooccipital junction** | | | | | | |
| A39c_l | -0.779 | 57 | 47 | 81 | Inferior Parietal Lobule | caudal area 39 |
| A7c_l | -0.685 | 76 | 56 | 58 | Superior Parietal Lobule | caudal area 7 |
| mOccG_l | -0.638 | 60 | 39 | 99 | Lateral Occipital Cortex | middle occipital gyrus |
| lsOccG_l | -0.626 | 69 | 50 | 74 | Lateral Occipital Cortex | lateral superior occipital gyrus |
| A7c_r | -0.592 | 110 | 58 | 56 | Superior Parietal Lobule | caudal area 7 |
| OPC_l | -0.584 | 73 | 29 | 108 | Lateral Occipital Cortex | occipital polar cortex |
| OPC_r | -0.577 | 113 | 31 | 107 | Lateral Occipital Cortex | occipital polar cortex |
| A7r_l | -0.574 | 75 | 67 | 47 | Superior Parietal Lobule | rostral area 7 |
| lsOccG_r | -0.557 | 120 | 53 | 74 | Lateral Occipital Cortex | lateral superior occipital gyrus |
| A7r_r | -0.553 | 111 | 70 | 45 | Superior Parietal Lobule | rostral area 7 |
| A39rd_l | -0.545 | 53 | 66 | 63 | Inferior Parietal Lobule | rostrodorsal area 39 |
| mOccG_r | -0.544 | 125 | 42 | 100 | Lateral Occipital Cortex | middle occipital gyrus |
| A7m_l | -0.519 | 86 | 63 | 59 | Precuneus | medial area 7 |
| A7m_r | -0.517 | 97 | 62 | 59 | Precuneus | medial area 7 |
| msOccG | -0.517 | 107 | 42 | 76 | Lateral Occipital Cortex | medial superior occipital gyrus |
| msOccG_l | -0.515 | 80 | 39 | 80 | Lateral Occipital Cortex | medial superior occipital gyrus |
| A39rd_r | -0.447 | 131 | 62 | 67 | Inferior Parietal Lobule | rostrodorsal area 39 |
| A39c_r | -0.445 | 136 | 56 | 90 | Inferior Parietal Lobule | caudal area 39 |
| A7pc_l | -0.445 | 69 | 79 | 45 | Superior Parietal Lobule | postcentral area 7 |
| **Component #10: Lateral frontoparietal lobes** | | | | | | |
| A4hf_r | -0.694 | 146 | 125 | 76 | Precentral Gyrus | area 4 |
| A40rd_r | -0.685 | 139 | 92 | 65 | Inferior Parietal Lobule | rostrodorsal area 40 |
| A2_r | -0.663 | 139 | 102 | 61 | Postcentral Gyrus | area 2 |
| A1_2_3ulhf_r | -0.600 | 141 | 112 | 65 | Postcentral Gyrus | area 1/2/3 |
| A40c_r | -0.548 | 149 | 83 | 72 | Inferior Parietal Lobule | caudal area 40 |
| A40rd_l | -0.540 | 40 | 94 | 67 | Inferior Parietal Lobule | rostrodorsal area 40 |
| A4hf_l | -0.515 | 42 | 120 | 69 | Precentral Gyrus | area 4 |
| A8vl_r | -0.513 | 133 | 154 | 69 | Middle Frontal Gyrus | ventrolateral area 8 |
| IFJ_r | -0.472 | 133 | 138 | 70 | Middle Frontal Gyrus | inferior frontal junction |
| A4ul_r | -0.469 | 125 | 107 | 103 | Precentral Gyrus | area 4 |
| A5l_r | -0.456 | 127 | 84 | 56 | Superior Parietal Lobule | lateral area 5 |
| A45c_r | -0.446 | 145 | 151 | 97 | Inferior Frontal Gyrus | caudal area 45 |
| A1_2_3ulhf_l | -0.427 | 42 | 111 | 65 | Postcentral Gyrus | area 1/2/3 |
| **Component #11: Medio ventral occipital cortex** | | | | | | |
| cCunG_r | -0.665 | 42 | 111 | 65 | Medio Ventral Occipital Cortex | caudal cuneus gyrus |
| cCunG_l | -0.653 | 85 | 34 | 110 | Medio Ventral Occipital Cortex | caudal cuneus gyrus |
| vmPOS_r | -0.648 | 105 | 64 | 98 | Medio Ventral Occipital Cortex | ventromesial parietooccipital sulcus |
| rCunG_r | -0.607 | 98 | 52 | 99 | Medio Ventral Occipital Cortex | rostral cuneus gyrus |
| cLinG_r | -0.527 | 101 | 43 | 119 | Medio Ventral Occipital Cortex | caudal lingual gyrus |
| vmPOS_l | -0.517 | 78 | 59 | 98 | Medio Ventral Occipital Cortex | ventromesial parietooccipital sulcus |
| rCunG_l | -0.484 | 86 | 47 | 100 | Medio Ventral Occipital Cortex | rostral cuneus gyrus |
| rLinG_r | -0.481 | 86 | 47 | 100 | Medio Ventral Occipital Cortex | rostral cuneus gyrus |
| msOccG_l | -0.473 | 80 | 38 | 80 | Lateral Occipital Cortex | medial superior occipital gyrus |
| cLinG_l | -0.436 | 80 | 46 | 121 | Medio Ventral Occipital Cortex | caudal lingual gyrus |
| iOccG_r | -0.421 | 123 | 44 | 122 | Lateral Occipital Cortex | inferior occipital gyrus |
| **Component #12: Superior and middle frontal gyrus** | | | | | | |
| A9l_l | -0.855 | 80 | 176 | 67 | Superior Frontal Gyrus | lateral area 9 |
| A9l_r | -0.842 | 104 | 175 | 67 | Superior Frontal Gyrus | lateral area 9 |
| A8dl_l | -0.738 | 73 | 150 | 55 | Superior Frontal Gyrus | dorsolateral area 8 |
| A8vl_l | -0.732 | 73 | 150 | 55 | Middle Frontal Gyrus | dorsolateral area 8 |
| A8dl_r | -0.728 | 113 | 152 | 56 | Superior Frontal Gyrus | dorsolateral area 8 |
| A9_46d_l | -0.614 | 64 | 170 | 76 | Middle Frontal Gyrus | dorsal area 9/46 |
| A9_46d_r | -0.581 | 122 | 164 | 72 | Middle Frontal Gyrus | dorsal area 9/46 |
| A6dl_r | -0.579 | 112 | 130 | 44 | Superior Frontal Gyrus | dorsolateral area 6 |
| A4t_l | -0.575 | 78 | 106 | 35 | Precentral Gyrus | area 4 |
| A46_l | -0.570 | 63 | 184 | 95 | Middle Frontal Gyrus | area 46 |
| A6vl_l | -0.563 | 59 | 130 | 53 | Middle Frontal Gyrus | ventrolateral area 6 |
| A6vl_r | -0.562 | 125 | 134 | 54 | Middle Frontal Gyrus | ventrolateral area 6 |
| A46_r | -0.559 | 119 | 182 | 90 | Middle Frontal Gyrus | area 46 |
| A6dl_l | -0.538 | 73 | 126 | 43 | Superior Frontal Gyrus | dorsolateral area 6 |
| A6cdl_l | -0.530 | 60 | 117 | 50 | Precentral Gyrus | caudal dorsolateral area 6 |
| A1_2_3tru_l | -0.508 | 70 | 92 | 41 | Postcentral Gyrus | area 1/2/3 |
| A7pc_l | -0.492 | 69 | 79 | 45 | Superior Parietal Lobule | postcentral area 7 |
| A7pc_r | -0.491 | 114 | 83 | 43 | Superior Parietal Lobule | postcentral area 7 |
| A4ul_l | -0.455 | 65 | 102 | 46 | Precentral Gyrus | area 4 |
| A1_2_3tru_r | -0.434 | 111 | 94 | 40 | Postcentral Gyrus | area 1/2/3 |
| **Component #13: Basal ganglia** | | | | | | |
| vmPu_l | -0.579 | 68 | 135 | 112 | Basal Ganglia | ventromedial putamen |
| NAC_l | -0.552 | 74 | 132 | 118 | Basal Ganglia | nucleus accumbens |
| lAmyg_l | -0.538 | 64 | 124 | 128 | Amygdala | lateral amygdala |
| dlPu_l | -0.514 | 63 | 123 | 107 | Basal Ganglia | dorsolateral putamen |
| dlPu_r | -0.507 | 120 | 124 | 107 | Basal Ganglia | dorsolateral putamen |
| GP_r | -0.503 | 113 | 126 | 105 | Basal Ganglia | globus pallidus |
| vmPu | -0.464 | 113 | 136 | 110 | Basal Ganglia | ventromedial putamen |
| MPMtha_r | -0.433 | 104 | 114 | 108 | Thalamus | pre-motor thalamus |
| A35_36r_l | -0.410 | 64 | 122 | 142 | Parahippocampus | rostral area 35/36 |
| mAmyg_l | -0.402 | 72 | 126 | 128 | Amygdala | medial amygdala |
| **Tab. 2 Regional components of PSP-RS patients defined by PCA** | | | | | | |
| **Regioncode** | **Factor loadings** | **X** | **Y** | **Z** | **Region** | **Subregion** |
| **Component #1: Right lateral temporal lobe** | | | | | | |
| A37vl_r | 0.748 | 145 | 71 | 118 | Inferior Temporal Gyrus | ventrolateral area 37 |
| cpSTS_r | 0.736 | 148 | 87 | 98 | Posterior Superior Temporal Sulcus | caudoposterior superior temporal sulcus |
| A20cl_r | 0.707 | 151 | 88 | 127 | Inferior Temporal Gyrus | caudolateral of area 20 |
| A21c_r | 0.689 | 155 | 99 | 123 | Middle Temporal Gyrus | caudal area 21 |
| rpSTS_r | 0.687 | 143 | 91 | 106 | Posterior Superior Temporal Sulcus | rostroposterior superior temporal sulcus |
| aSTS_r | 0.680 | 149 | 112 | 119 | Middle Temporal Gyrus | anterior superior temporal sulcus |
| V5_MT_plus_r | 0.648 | 139 | 58 | 111 | Lateral Occipital Cortex | area V5/MT+ |
| A37dl_r | 0.631 | 151 | 74 | 107 | Middle Temporal Gyrus | dorsolateral area 37 |
| A20cv_r | 0.627 | 145 | 97 | 135 | Inferior Temporal Gyrus | caudoventral of area 20 |
| A20r_r | 0.586 | 131 | 129 | 152 | Inferior Temporal Gyrus | rostral area 20 |
| A37lv_r | 0.581 | 133 | 79 | 129 | Fusiform Gyrus | ventrolateral area 37 |
| A20il_r | 0.565 | 145 | 117 | 141 | Inferior Temporal Gyrus | intermediate lateral area 20 |
| A22r_R | 0.561 | 147 | 116 | 114 | Superior Temporal Gyrus | rostral area 22 |
| A37elv_r | 0.551 | 144 | 76 | 129 | Inferior Temporal Gyrus | extreme lateroventral area 37 |
| vId_vIg_r | 0.547 | 130 | 126 | 118 | Insular Gyrus | ventral granular insular |
| iOCCG_l | 0.539 | 60 | 41 | 123 | Lateral Occipital Cortex | inferior occipital gyrus |
| V5_MT_plus_l | 0.535 | 45 | 54 | 107 | Lateral Occipital Cortex | area V5/MT+ |
| A40rv_r | 0.524 | 146 | 101 | 84 | Inferior Parietal Lobule | rostroventral area 40 |
| A41_42_r | 0.519 | 145 | 104 | 99 | Superior Temporal Gyrus | area 41/42 |
| dId_r | 0.512 | 129 | 133 | 104 | Insular Gyrus | dorsal dysgranular insular |
| A39rv_r | 0.503 | 144 | 73 | 86 | Inferior Parietal Lobule | rostroventral area 39 |
| A20iv_r | 0.495 | 137 | 115 | 142 | Inferior Temporal Gyrus | intermediate ventral area 20 |
| A22c_r | 0.494 | 157 | 107 | 103 | Superior Temporal Gyrus | caudal area 22 |
| A37elv_l | 0.489 | 40 | 71 | 124 | Inferior Temporal Gyrus | extreme lateroventral area 37 |
| G_r | 0.487 | 128 | 110 | 101 | Insular Gyrus | hypergranular insular |
| A37mv_r | 0.472 | 122 | 67 | 124 | Fusiform Gyrus | medioventral area 37 |
| A1_2_3tonIa_r | 0.471 | 147 | 117 | 94 | Postcentral Gyrus | area 1/2/3 |
| dIg_r | 0.460 | 130 | 121 | 101 | Insular Gyrus | dorsal granular insular |
| cLinG_l | 0.442 | 80 | 46 | 121 | Medio Ventral Occipital Cortex | caudal lingual gyrus |
| mOccG_r | 0.418 | 125 | 42 | 100 | Lateral Occipital Cortex | middle occipital gyrus |
| rcunG_l | 0.417 | 86 | 47 | 100 | Medio Ventral Occipital Cortex | rostral cuneus gyrus |
| lsOCCG_r | 0.407 | 120 | 53 | 74 | Lateral Occipital Cortex | lateral superior occipital gyrus |
| A10l_r | 0.402 | 117 | 189 | 111 | Middle Frontal Fyrus | lateral area 10 |
| **Component #2: Mesial frontoparietal lobes** | | | | | | |
| A1_2_3ll_l | 0.772 | 84 | 89 | 51 | Paracentral Lobule | area 1/2/3 |
| A1_2_3_ll_r | 0.733 | 102 | 92 | 55 | Paracentral Lobule | area 1/2/3 |
| A4ul_l | 0.728 | 65 | 102 | 46 | Precentral Gyrus | area 4 |
| A4ll_r | 0.698 | 96 | 105 | 48 | Paracentral Lobule | area 4 |
| A4ll_l | 0.673 | 87 | 104 | 48 | Paracentral Lobule | area 4 |
| A4t_r | 0.648 | 106 | 105 | 38 | Precentral Gyrus | area 4 |
| A1_2_3tru_r | 0.600 | 111 | 94 | 40 | Postcentral Gyrus | area 1/2/3 |
| A4ul_r | 0.580 | 125 | 107 | 51 | Precentral Gyrus | area 4 |
| A6dl_l | 0.556 | 73 | 126 | 43 | Superior Frontal Gyrus | dorsolateral area 6 |
| A6dl_r | 0.539 | 112 | 130 | 44 | Superior Frontal Gyrus | dorsolateral area 6 |
| A1_2_3tru_l | 0.536 | 70 | 92 | 41 | Postcentral Gyrus | area 1/2/3 |
| A6vl_r | 0.523 | 125 | 134 | 54 | Middle Frontal Gyrus | ventrolateral area 6 |
| A6cdl_l | 0.518 | 60 | 117 | 50 | Precentral Gyrus | caudal corsolateral area 6 |
| A6vl_l | 0.515 | 59 | 130 | 53 | Middle Frontal Gyrus | ventrolateral area 6 |
| A5m_l | 0.510 | 83 | 79 | 52 | Precuneus | medial area 5 |
| A4t_l | 0.500 | 78 | 106 | 35 | Precentral Gyrus | area 4 |
| A5m_r | 0.492 | 99 | 80 | 51 | Precuneus | medial area 5 |
| rCunG_r | 0.485 | 98 | 52 | 99 | Medio Ventral Occipital Cortex | rostral cuneus gyrus |
| A6m_r | 0.459 | 99 | 123 | 49 | Superior Frontal Gyrus | medial area 6 |
| A40c_r | 0.444 | 149 | 83 | 72 | Inferior Parietal Lobule | caudal area 40 |
| A1_2_3ulhf_l | 0.435 | 42 | 111 | 65 | Postcentral Gyrus | area 1/2/3 |
| A8m_r | 0.430 | 99 | 143 | 54 | Superior Frontal Gyrus | medial area 8 |
| A40rd_r | 0.415 | 139 | 92 | 65 | Inferior Parietal Lobule | rostrodorsal area 40 |
| A23c_l | 0.411 | 84 | 104 | 68 | Cingulate Gyrus | caudal area 23 |
| **Component #3: Caudate nucleus/thalamus** | | | | | | |
| cTtha_l | 0.987 | 80 | 106 | 96 | Thalamus | caudal temporal thalamus |
| cTtha_r | 0.980 | 101 | 113 | 95 | Thalamus | caudal temporal thalamus |
| dCa_r | 0.917 | 105 | 133 | 94 | Basal Ganglia | dorsal caudate |
| dCa_l | 0.873 | 77 | 130 | 92 | Basal Ganglia | dorsal caudate |
| rTtha_l | 0.858 | 84 | 114 | 102 | Thalamus | rostral temporal thalamus |
| rTtha_r | 0.854 | 94 | 115 | 103 | Thalamus | rostral temporal thalamus |
| Otha_r | 0.843 | 104 | 101 | 102 | Thalamus | occipital thalamus |
| lPFtha_r | 0.822 | 103 | 111 | 101 | Thalamus | lateral pre-frontal thalamus |
| mPFtha_r | 0.788 | 98 | 117 | 103 | Thalamus | medial pre-frontal thalamus |
| PPtha_r | 0.719 | 106 | 103 | 103 | Thalamus | posterior parietal thalamus |
| vCa_l | 0.674 | 79 | 142 | 108 | Basal Ganglia | ventral caudate |
| PPtha_l | 0.665 | 75 | 104 | 103 | Thalamus | posterior parietal thalamus |
| A24rv_r | 0.647 | 96 | 149 | 96 | Cingulate Cortex | rostroventral area 24 |
| vCa_r | 0.614 | 106 | 142 | 111 | Basal Ganglia | ventral caudate |
| mPFtha_l | 0.588 | 84 | 116 | 103 | Thalamus | medial pre-frontal thalamus |
| Otha_l | 0.534 | 76 | 100 | 105 | Thalamus | occipital thalamus |
| **Component #4: Parahippocampal gyrus** | | | | | | |
| A28_34_r | 0.808 | 109 | 118 | 138 | Parahippocampal Gyrus | area 28/34 |
| A28_34_l | 0.659 | 72 | 117 | 139 | Parahippocampal Gyrus | area 28/34 |
| A35_36c_r | 0.629 | 117 | 105 | 136 | Parahippocampal Gyrus | caudal area 35/36 |
| rHipp_r | 0.605 | 113 | 116 | 129 | Hippocampus | rostral hippocampus |
| TI_l | 0.580 | 68 | 131 | 140 | Parahippocampal Gyrus | temporal agranular insular cortex |
| mAmyg_r | 0.575 | 110 | 126 | 128 | Amygdala | medial amygdala |
| TH_r | 0.531 | 109 | 92 | 121 | Parahippocampal Gyrus | area TH |
| TI_r | 0.488 | 112 | 130 | 145 | Parahippocampal Gyrus | temporal agranular insular cortex |
| rHipp_l | 0.466 | 69 | 114 | 127 | Hippocampus | rostral hippocampus |
| mAmgy_l | 0.465 | 72 | 126 | 128 | Amygdala | medial amygdala |
| **Component #5: Parietooccipital junction** | | | | | | |
| lsOccG_l | -0.833 | 69 | 50 | 74 | Lateral Occipital Cortex | lateral superior occipital gyrus |
| A7c_l | -0.814 | 76 | 56 | 58 | Superior Parietal Lobule | caudal area 7 |
| A7c_r | -0.793 | 110 | 58 | 56 | Superior Parietal Lobule | caudal area 7 |
| mOccG_r | -0.776 | 107 | 42 | 76 | Lateral Occipital Cortex | medial superior occipital gyrus |
| A39c_l | -0.776 | 57 | 47 | 81 | Inferior Parietal Lobule | caudal area 39 |
| msOccG_l | -0.699 | 80 | 39 | 80 | Lateral Occipital Cortex | medial superior occipital gyrus |
| A7r_l | -0.621 | 75 | 67 | 47 | Superior Parietal Lobule | rostral area 7 |
| A7m_r | -0.595 | 97 | 62 | 59 | Precuneus | medial area 7 |
| A39rd_r | -0.584 | 131 | 62 | 67 | Inferior Parietal Lobule | rostrodorsal area 39 |
| A39rd_l | -0.549 | 53 | 66 | 63 | Inferior Parietal Lobule | rostrodorsal area 39 |
| A7r_r | -0.546 | 111 | 70 | 45 | Superior Parietal Lobule | rostral area 7 |
| A7m_l | -0.531 | 86 | 63 | 59 | Precuneus | medial area 7 |
| cCunG_l | -0.529 | 85 | 34 | 110 | Medio Ventral Occipital Cortex | caudal cuneus gyrus |
| mOccG_l | -0.503 | 60 | 39 | 99 | Lateral Occipital Cortex | middle Occipital Gyrus |
| lsOccG_r | -0.502 | 120 | 53 | 74 | Lateral Occipital Cortex | lateral superior occipital gyrus |
| mOccG_r | -0.502 | 125 | 42 | 100 | Lateral Occipital Cortex | middle occipital gyrus |
| OPC_r | -0.498 | 113 | 31 | 107 | Lateral Occipital Cortex | occipital polar cortex |
| A7pc_l | -0.498 | 69 | 79 | 45 | Superior Parietal Lobule | postcentral area 7 |
| A39c_r | -0.484 | 136 | 56 | 90 | Inferior Parietal Lobule | caudal area 39 |
| cCunG_r | -0.484 | 99 | 38 | 98 | Medio Ventral Occipital Cortex | caudal cuneus gyrus |
| OPC_l | -0.471 | 73 | 29 | 108 | Lateral Occipital Cortex | occipital polar cortex |
| A7ip_l | -0.427 | 64 | 68 | 56 | Superior Parietal Lobule | intraparietal area 7 |
| A7pc_r | -0.414 | 114 | 83 | 43 | Superior Parietal Lobule | postcentral area 7 |
| rCunG_l | -0.403 | 86 | 47 | 100 | Medio Ventral Occipital Cortex | rostral cuneus gyrus |
| **Component #6: Lateral frontoparietal lobes** | | | | | | |
| A4hf_r | -0.744 | 146 | 125 | 76 | Precentral Gyrus | area 4 |
| A8vl_r | -0.715 | 133 | 154 | 69 | Middle Frontal Gyrus | ventrolateral area 8 |
| A2_r | -0.661 | 139 | 102 | 61 | Postcentral Gyrus | area 2 |
| A40rd_r | -0.592 | 139 | 92 | 65 | Inferior Parietal Lobule | rostrodorsal area 40 |
| A1_2_3ulhf_r | -0.577 | 139 | 92 | 65 | Postcentral Gyrus | rostrodorsal area 40 |
| A4hf_l | -0.548 | 42 | 120 | 69 | Precentral Gyrus | area 4 |
| A40c_r | -0.529 | 149 | 83 | 72 | Inferior Parietal Lobule | caudal area 40 |
| IFJ_r | -0.490 | 133 | 138 | 70 | Middle Frontal Gyrus | inferior frontal junction |
| IFH_l | -0.434 | 50 | 141 | 72 | Middle Frontal Gyrus | inferior frontal junction |
| A4ul_r | -0.430 | 125 | 107 | 51 | Precentral Gyrus | area 4 |
| A45c_r | -0.418 | 145 | 151 | 97 | Inferior Frontal Gyrus | caudal area 45 |
| **Component #7: Hippocampus/thalamus** | | | | | | |
| TL_l | 0.819 | 63 | 97 | 127 | Parahippocampal Gyrus | posterior parahippocampal gyrus |
| TL_l | 0.810 | 63 | 97 | 127 | Parahippocampal Gyrus | posterior parahippocampal gyrus |
| A35_36c_l | 0.737 | 66 | 104 | 135 | Parahippocampal Gyrus | caudal area 35/36 |
| Stha_r | 0.733 | 108 | 106 | 106 | Thalamus | sensory thalamus |
| TL_r | 0.662 | 120 | 98 | 127 | Parahippocampal Gyrus | posterior parahippocampal gyrus |
| A35_36r_l | 0.658 | 64 | 122 | 142 | Parahippocampal Gyrus | rostral area 35/36 |
| cHipp_l | 0.623 | 63 | 98 | 119 | Hippocampus | caudal hippocampus |
| Stha_l | 0.569 | 73 | 105 | 105 | Thalamus | sensory thalamus |
| cHipp_r | 0.563 | 120 | 101 | 120 | Hippocampus | caudal hippocampus |
| rLinG_l | 0.559 | 74 | 68 | 116 | Medio Ventral Occipital Cortex | rostral lingual gyrus |
| IPPFtha_l | 0.530 | 74 | 68 | 116 | Thalamus | lateral pre-frontal thalamus |
| Otha_l | 0.509 | 76 | 100 | 105 | Thalamus | occipital thalamus |
| A20rv_l | 0.479 | 58 | 112 | 141 | Fusiform Gyrus | rostroventral area 20 |
| mPFtha_l | 0.461 | 84 | 116 | 103 | Thalamus | medial pre-frontal thalamus |
| PPtha_l | 0.446 | 75 | 104 | 103 | Thalamus | posterior parietal thalamus |
| **Component #8 Left parietotemporal junction** | | | | | | |
| TE1.0_TE1.2_l | 0.825 | 41 | 117 | 107 | Superior Temporal Gyrus | TE1.0 and TE1.2 |
| A22r_l | 0.722 | 36 | 125 | 118 | Superior Temporal Gyrus | rostral area 22 |
| A1_2_3tonIa_l | 0.577 | 35 | 114 | 92 | Postcentral Gyrus | area 1/2/3 |
| aSTS_l | 0.566 | 32 | 109 | 118 | Middle Temporal Gyrus | anterior superior temporal sulcus |
| G_l | 0.550 | 55 | 108 | 99 | Insular Gyrus | hypergranular insular |
| A4tl_l | 0.548 | 39 | 128 | 100 | Precentral Gyrus | area 4 |
| A1_2_3ulhf_l | 0.513 | 42 | 111 | 65 | Postcentral Gyrus | area 1/2/3 |
| A41_42_l | 0.509 | 37 | 96 | 96 | Superior Temporal Gyrus | area 41/42 |
| A21r_l | 0.477 | 38 | 131 | 137 | Middle Temporal Gyrus | rostral area 21 |
| rpSTS_l | 0.473 | 37 | 88 | 105 | Posterior Superior Temporal Sulcus | rostroposterior superior temporal sulcus |
| mPMtha_r | 0.459 | 104 | 114 | 108 | Thalamus | pre-motor thalamus |
| A21c_l | 0.430 | 26 | 98 | 120 | Middle Temporal Gyrus | caudal area 21 |
| dId_l | 0.402 | 53 | 133 | 104 | Insular Gyrus | dorsal dysgranular insular |
| **Component #9: Basal Ganglia** | | | | | | |
| NAC_l | -0.765 | 74 | 132 | 118 | Basal Ganglia | nucleus accumbens |
| vmPu_l | -0.761 | 68 | 135 | 112 | Basal Ganglia | ventromedial putamen |
| dlPu_r | -0.749 | 120 | 124 | 107 | Basal Ganglia | dorsolateral putamen |
| dlPu_l | -0.747 | 63 | 123 | 107 | Basal Ganglia | dorsolateral putamen |
| vmPu_r | -0.675 | 113 | 136 | 110 | Basal ganglia | ventromedial putamen |
| GP_l | -0.675 | 69 | 126 | 105 | Basal Ganglia | globus pallidus |
| A13_l | -0.592 | 81 | 146 | 127 | Orbital Gyrus | area 13 |
| lAmyg_l | -0.570 | 64 | 124 | 128 | Amygdala | lateral amygdala |
| dId_l | -0.570 | 53 | 133 | 104 | Insular Gyrus | dorsal dysgraunar insular |
| GP_r | -0.561 | 42 | 133 | 104 | Basal Ganglia | globus pallidus |
| dIa_r | -0.555 | 128 | 146 | 108 | Insular Gyrus | dorsal agranular insular |
| A11l_l | -0.542 | 68 | 166 | 125 | Orbital Gyrus | lateral area 11 |
| A13_r | -0.532 | 100 | 149 | 127 | Orbital Gyrus | area 13 |
| mAmyg_l | -0.510 | 72 | 126 | 128 | Amygdala | medial amygdala |
| vIa_r | -0.508 | 124 | 142 | 121 | Insular Gyrus | ventral agranular insular |
| NAC_r | -0.500 | 105 | 136 | 117 | Basal Ganglia | nucleus accumbens |
| A44op_l | -0.487 | 52 | 151 | 104 | Inferior Frontal Gyrus | opercular area 44 |
| A11l_r | -0.486 | 114 | 164 | 126 | Orbital Gyrus | lateral area 11 |
| A44op_r | -0.458 | 133 | 150 | 105 | Inferior Frontal Gyrus | opercular area 44 |
| cCunG_r | -0.438 | 99 | 38 | 98 | Medio Ventral Occipital Cortex | caudal cuneus gyrus |
| dIa_l | -0.436 | 57 | 146 | 106 | Insular Gyrus | dorsal agranular insular |
| lAmyg_r | -0.418 | 118 | 125 | 128 | Amygdala | lateral amygdala |
| **Component #10: Superior and middle frontal gyrus** | | | | | | |
| A9l_l | -0.882 | 80 | 176 | 67 | Superior Frontal Gyrus | lateral area 9 |
| A9l_r | -0.722 | 104 | 175 | 67 | Superior Frontal Gyrus | lateral area 9 |
| A8dl_l | -0.670 | 73 | 150 | 55 | Superior Frontal Gyrus | dorsolateral area 8 |
| A46_l | -0.666 | 73 | 150 | 55 | Middle Frontal Gyrus | dorsolateral area 8 |
| A8vl_l | -0.647 | 59 | 150 | 62 | Middle Frontal Gyrus | ventrolateral area 8 |
| A9_46d_l | -0.633 | 64 | 179 | 76 | Middle Frontal Gyrus | dorsal area 9/46 |
| A46_r | -0.590 | 119 | 182 | 90 | Middle Frontal Gyrus | area 46 |
| A8dl_r | -0.585 | 113 | 152 | 56 | Superior Frontal Gyrus | dorsolateral area 8 |
| A9_46d_r | -0.576 | 122 | 164 | 72 | Middle Frontal Gyrus | dorsal area 9/46 |
| A6vl_l | -0.538 | 59 | 130 | 53 | Middle Frontal Gyrus | ventrolateral area 6 |
| A9_46v_l | -0.453 | 50 | 168 | 91 | Middle Frontal Gyrus | ventral area 9/46 |
| A10l_r | -0.430 | 117 | 189 | 111 | Middle Frontal Gyrus | lateral area 10 |
| A10l_l | -0.424 | 66 | 189 | 112 | Middle Frontal Gyrus | lateral area 10 |
| A9_46v_r | -0.409 | 133 | 172 | 94 | Middle Frontal Gyrus | ventral area 9/46 |
| **Component #11: Inferolateral frontal lobe** | | | | | | |
| A44d_l | 0.453 | 45 | 141 | 84 | Inferior Frontal Gyrus | dorsal area 44 |
| A45c_r | 0.432 | 145 | 151 | 97 | Inferior Frontal Gyrus | caudal area 45 |
| mPMtha_r | 0.432 | 104 | 114 | 108 | Thalamus | pre-motor thalamus |
| A45r_l | 0.418 | 42 | 164 | 110 | Inferior Frontal Gyrus | rostral area 45 |
| **Component #12: Medial superior frontal gyrus/anterior cingulate cortex/orbital gyrus** | | | | | | |
| A24cd_r | -0.795 | 95 | 133 | 70 | Cingulate Gyrus | caudodorsal area 24 |
| A24cd_l | -0.744 | 86 | 134 | 71 | Cingulate Gyrus | caudodorsal area 24 |
| A32p_r | -0.744 | 96 | 155 | 80 | Cingulate Gyrus | pregenual area 32 |
| A32p_l | -0.730 | 85 | 162 | 87 | Cingulate Gyrus | pregenual area 32 |
| A8m_l | -0.673 | 87 | 142 | 54 | Superior Frontal Gyrus | medial area 8 |
| A9m_l | -0.629 | 87 | 142 | 54 | Superior Frontal Gyrus | medial area 8 |
| A32sg_r | -0.617 | 96 | 169 | 101 | Cingulate Gyrus | subgenual area 32 |
| A9m_r | -0.616 | 97 | 165 | 73 | Superior Frontal Gyrus | medial area 9 |
| A8m_r | -0.609 | 99 | 143 | 54 | Superior Frontal Gyrus | medial area 8 |
| A6m_l | -0.556 | 85 | 122 | 51 | Superior Frontal Gyrus | medial area 6 |
| A10m_r | -0.537 | 99 | 186 | 94 | Superior Frontal Gyrus | medial area 10 |
| A24rv_l | -0.527 | 88 | 136 | 84 | Cingulate Gyrus | rostroventral area 24 |
| A10m_l | -0.490 | 84 | 183 | 92 | Superior Frontal Gyrus | medial area 10 |
| A6m_r | -0.485 | 99 | 123 | 49 | Superior Frontal Gyrus | medial area 6 |
| A23d_r | -0.420 | 96 | 90 | 78 | Cingulate Gyrus | dorsal area 23 |
| **Component #13: Superior Temporal Lobe** | | | | | | |
| A38m_r | -0.820 | 122 | 144 | 142 | Superior Temporal Gyrus | medial area 38 |
| A38m_l | -0.798 | 59 | 143 | 142 | Superior Temporal Lobe | medial area 38 |
| A38l_l | -0.712 | 46 | 139 | 127 | Superior Temporal Lobe | lateral area 38 |
| A38l_r | -0.659 | 138 | 141 | 128 | Superior Temporal Lobe | lateral area 38 |
| A12_47l_l | -0.564 | 50 | 160 | 117 | Orbital Gyrus | lateral area 12/47 |
| A11m_r | -0.553 | 98 | 185 | 124 | Orbital Gyrus | medial area 11 |
| A45r_l | -0.520 | 42 | 164 | 110 | Inferior Frontal Gyrus | rostral area 45 |
| A10m_r | -0.457 | 99 | 186 | 94 | Superior Frontal Gyrus | medial area 10 |
| A23v_r | -0.451 | 99 | 84 | 98 | Cingulate Gyrus | ventral area 23 |
| A21r_r | -0.422 | 142 | 134 | 141 | Middle Temporal Gyrus | rostral area 21 |
| TI_r | -0.405 | 112 | 130 | 145 | Parahippocampal gyrus | temporal agranular insular cortex |

| **Tab. 3 Regional components of CBS patients defined by PCA** | | | | | | |
| --- | --- | --- | --- | --- | --- | --- |
| **Regioncode** | **Factor loadings** | **X** | **Y** | **Z** | **Region** | **Subregion** |
| **Component #1: Temporooccipital Cortex** | | | | | | |
| iOccG_r | 0.670 | 123 | 144 | 122 | Lateral Occipital Cortex | inferior occipital gyrus |
| V5_MT_plus_r | 0.657 | 139 | 58 | 111 | Lateral Occipital Cortex | area V5/MT+ |
| Occipital Lobe | 0.640 | 101 | 43 | 119 | Medio Ventral Occipital Cortex | caudal lingual gyrus |
| cpSTS_l | 0.613 | 39 | 78 | 98 | Posterior Superior Temporal Sulcus | caudoposterior superior temporal sulcus |
| iOCCG_l | 0.607 | 60 | 41 | 123 | Lateral Occipital Cortex | inferior occipital gyrus |
| A37dl_r | 0.550 | 151 | 74 | 107 | Middle Temporal Gyrus | dorsolateral area 37 |
| A37mv_r | 0.539 | 122 | 67 | 124 | Fusiform Gyrus | medioventral area 37 |
| A23c_l | 0.507 | 84 | 104 | 68 | Cingulate Gyrus | caudal area 23 |
| mOccG_r | 0.470 | 125 | 42 | 100 | Lateral Occipital Cortex | middle occipital gyrus |
| A37mv_l | 0.447 | 60 | 64 | 124 | Fusiform Gyrus | medioventral area 37 |
| A7ip_r | 0.423 | 122 | 72 | 57 | Superior Parietal Lobule | intraparietal area 7 |
| **Component #2: Hippocampus/thalamus** | | | | | | |
| TL_l | 0.825 | 63 | 97 | 127 | Parahippocampal Gyrus | posterior parahippocampal gyrus |
| cHipp_l | 0.769 | 63 | 98 | 119 | Hipocampus | caudal hippocampus |
| A23v_l | 0.617 | 83 | 80 | 100 | Cingulate Gyrus | ventral area 23 |
| rLinG_l | 0.583 | 74 | 68 | 116 | Medio Ventral Occipital Cortex | rostral lingual gyrus |
| A20rv_l | 0.541 | 58 | 112 | 141 | Fusiform Gyrus | rostroventral area 20 |
| cHipp_r | 0.537 | 120 | 101 | 120 | Hippocampus | caudal hippocampus |
| TL_r | 0.528 | 120 | 98 | 127 | Parahippocampal Gyrus | posterior parahippocampal gyrus |
| A35_35c_l | 0.520 | 66 | 104 | 135 | Parahippocampal Gyrus | caudal area 35/36 |
| TH_l | 0.500 | 74 | 89 | 119 | Parahippocampal Gyrus | area TH |
| A23v_r | 0.473 | 99 | 84 | 98 | Cingulate Gyrus | ventral area 23 |
| A35_36r_l | 0.433 | 64 | 122 | 142 | Parahippocampal Gyrus | rostral area 35/36 |
| A37mv_l | 0.416 | 60 | 64 | 124 | Fusiform Gyrus | medioventral area 37 |
| Otha_l | 0.411 | 76 | 100 | 105 | Thalamus | occipital thalamus |
| Stha_l | 0.401 | 73 | 105 | 105 | Thalamus | sensory thalamus |
| **Component #3: caudate nucleus/thalamus** | | | | | | |
| dCa_r | 0.975 | 105 | 133 | 94 | Basal Ganglia | dorsal caudate |
| rTtha_r | 0.955 | 94 | 115 | 103 | Thalamus | rostral temporal thalamus |
| cTtha_r | 0.942 | 101 | 113 | 95 | Thalamus | caudal temporal thalamus |
| rTtha_l | 0.938 | 84 | 114 | 102 | Thalamus | rostral temporal thalamus |
| dCa_l | 0.934 | 77 | 130 | 92 | Basal Ganglia | dorsal caudate |
| cTtha_l | 0.907 | 80 | 106 | 96 | Thalamus | caudal temporal thalamus |
| mPFtha_r | 0.902 | 98 | 117 | 103 | Thalamus | medial pre-frontal thalamus |
| Otha_r | 0.823 | 104 | 101 | 102 | Thalamus | occipital thalamus |
| mPFtha_l | 0.783 | 84 | 116 | 103 | Thalamus | medial pre-frontal thalamus |
| lPFtha_r | 0.736 | 103 | 111 | 102 | Thalamus | lateral pre-frontal thalamus |
| PPtha_r | 0.686 | 106 | 103 | 103 | Thalamus | posterior parietal thalamus |
| vCa_l | 0.663 | 79 | 142 | 108 | Basal Ganglia | ventral caudate |
| PPtha_l | 0.577 | 75 | 104 | 103 | Thalamus | posterior parietal thalamus |
| vCa_r | 0.548 | 106 | 142 | 111 | Basal Ganglia | ventral caudate |
| A24rv_r | 0.525 | 96 | 149 | 96 | Cingulate Gyrus | rostroventral area 24 |
| Otha_l | 0.468 | 76 | 100 | 105 | Thalamus | occipital thalamus |
| **Component #4: Superior Temporal Gyrus** | | | | | | |
| A38l_r | 0.902 | 46 | 139 | 127 | Superior Temporal Gyrus | lateral area 38 |
| A38m_r | 0.695 | 122 | 144 | 142 | Superior Temporal Gyrus | medial area 38 |
| A38l_l | 0.515 | 46 | 139 | 127 | Superior Temporal Gyrus | lateral area 38 |
| A22r_r | 0.493 | 147 | 116 | 114 | Superior Temporal Gyrus | rostral area 22 |
| TH_r | 0.466 | 109 | 92 | 121 | Parahippocampal Gyrus | area TH |
| TH_l | 0.452 | 74 | 89 | 119 | Parahippocampal Gyrus | area TH |
| **Component #5: Left parietotemporal junction** | | | | | | |
| A42_42_l | -0.816 | 37 | 96 | 96 | Superior Temporal Gyrus | area 41/42 |
| A40rv_l | -0.693 | 37 | 97 | 86 | Inferior Parietal Lobule | rostroventral area 40 |
| A41_42_r | -0.667 | 145 | 104 | 99 | Superior Temporal Gyrus | area 41/42 |
| TE1.0_TE1.2_l | -0.661 | 41 | 117 | 107 | Superior Temporal Gyrus | TE1.0 and TE1.2 |
| A1_2_3tonIa_l | -0.660 | 35 | 114 | 92 | Postcentral Gyrus | area 1/2/3 |
| IJF_l | -0.551 | 50 | 141 | 72 | Middle Frontal Gyrus | inferior frontal junction |
| G_l | -0.543 | 55 | 108 | 99 | Insular Gyrus | hypergranular insular |
| **Component #6: Superior and middle frontal gyrus** | | | | | | |
| A9l_l | -0.893 | 80 | 176 | 67 | Superior Frontal Gyrus | lateral area 9 |
| A9l_r | -0.888 | 104 | 175 | 67 | Superior Frontal Gyrus | lateral area 9 |
| A6dl_r | -0.839 | 112 | 130 | 44 | Superior Frontal Gyrus | dorsolateral area 6 |
| A9dl_r | -0.818 | 113 | 152 | 56 | Superior Frontal Gyrus | dorsolateral area 8 |
| A8dl_l | -0.786 | 73 | 150 | 55 | Superior Frontal Gyrus | dorsolateral area 8 |
| A6dl_l | -0.765 | 73 | 126 | 43 | Superior Frontal Gyrus | dorsolateral area 6 |
| A8vl_l | -0.708 | 59 | 150 | 62 | Middle Frontal Gyrus | ventrolateral area 8 |
| A4ul_r | -0.708 | 125 | 107 | 51 | Precentral Gyrus | area 4 |
| A4t_l | -0.696 | 78 | 106 | 35 | Precentral Gyrus | area 4 |
| A6vl_r | -0.681 | 125 | 134 | 54 | Middle Frontal Gyrus | ventrolateral area 6 |
| A4t_r | -0.663 | 106 | 105 | 38 | Precentral Gyrus | area 4 |
| A6vl_l | -0.660 | 59 | 130 | 53 | Middle Frontal Gyrus | ventrolateral area 6 |
| A6cdl_l | -0.629 | 60 | 117 | 50 | Precentral Gyrus | caudal dorsolateral area 6 |
| A4ul_l | -0.628 | 65 | 102 | 46 | Precentral Gyrus | area 4 |
| A2_l | -0.628 | 46 | 97 | 59 | Postcentral Gyrus | area 2 |
| A1_2_3tru_l | -0.605 | 70 | 92 | 41 | Postcentral Gyrus | area 1/2/3 |
| A7pc_r | -0.603 | 114 | 84 | 43 | Superior Parietal Lobule | postcentral area 7 |
| A40rd_l | -0.599 | 40 | 94 | 67 | Inferior Parietal Lobule | rostrodorsal area 40 |
| A1_2_3tru_r | -0.594 | 111 | 94 | 40 | Postcentral Gyrus | area 1/2/3 |
| A9_46d_l | -0.592 | 64 | 170 | 76 | Middle Frontal Gyrus | dorsal area 9/46 |
| A8m_r | -0.573 | 99 | 143 | 54 | Superior Frontal Gyrus | medial area 8 |
| A6m_r | -0.543 | 99 | 123 | 49 | Superior Frontal Gyrus | medial area 6 |
| A7pc_l | -0.539 | 69 | 79 | 45 | Superior Parietal Lobule | postcentral area 7 |
| A8m_l | -0.535 | 87 | 142 | 54 | Superior Frontal Gyrus | medial area 8 |
| lsOccG_l | -0.474 | 69 | 50 | 74 | Lateral Occipital Cortex | lateral superior occipital gyrus |
| A9m_l | -0.423 | 85 | 122 | 51 | Superior Frontal Gyrus | medial area 6 |
| A6m_l | -0.412 | 85 | 122 | 51 | Superior Frontal Gyrus | medial area 6 |
| **Component #7: Parahippocampal Gyrus** | | | | | | |
| TI_r | 0.846 | 112 | 130 | 145 | Parahippocampal Gyrus | area TI |
| TI_l | 0.724 | 68 | 131 | 140 | Parahippocampal Gyrus | area TI |
| A38m_l | 0.624 | 59 | 143 | 142 | Superior Temporal Gyrus | medial area 38 |
| A28_34_r | 0.593 | 109 | 118 | 138 | Parahippocampal Gyrus | area 28/34 |
| A28_34_l | 0.526 | 72 | 117 | 139 | Parahippocampal Gyrus | area 28/34 |
| A35_36r_l | 0.486 | 64 | 122 | 142 | Parahippocampal Gyrus | rostral area 35/36 |
| TH_r | 0.427 | 109 | 92 | 121 | Parahippocampal Gyrus | area TH |
| **Component #8: Parietooccipital junction** | | | | | | |
| A39c_l | 0.718 | 57 | 47 | 81 | Inferior Parietal Lobule | caudal area 39 |
| A5l_r | 0.623 | 127 | 84 | 56 | Superior Parietal Lobule | lateral area 5 |
| mOccG_l | 0.583 | 60 | 39 | 99 | Lateral Occipital Cortex | middle occipital gyrus |
| msOccG_l | 0.529 | 80 | 39 | 80 | Lateral Occipital Cortex | medial superior occipital gyrus |
| lsOccG_r | 0.529 | 120 | 53 | 74 | Lateral Occipital Cortex | lateral superior occipital gyrus |
| A7pc_l | 0.438 | 69 | 79 | 45 | Superior Parietal Lobule | postcentral area 7 |
| lsOccG_l | 0.434 | 69 | 50 | 74 | Lateral Occipital Cortex | lateral superior occipital gyrus |
| A7m_r | 0.425 | 97 | 62 | 59 | Precuneus | medial area 7 |
| OPC_l | 0.420 | 73 | 29 | 108 | Lateral Occipital Cortex | occipital polar cortex |
| A7pc_r | 0.412 | 114 | 83 | 43 | Superior Parietal Lobule | postcentral area 7 |
| OPC_r | 0.407 | 113 | 31 | 107 | Lateral Occipital Cortex | occipital polar cortex |
| **Component #9: mesial frontoparietal lobes** | | | | | | |
| A1_2_3ll_r | 0.786 | 101 | 92 | 55 | Paracentral Lobule | area 1/2/3 |
| A1_2_3ll_l | 0.742 | 84 | 89 | 51 | Paracentral Lobule | area 1/2/3 |
| A5m_l | 0.729 | 83 | 79 | 52 | Precuneus | medial area 5 |
| A23c_r | 0.686 | 97 | 107 | 69 | Cingulate Gyrus | caudal area 23 |
| A5m_r | 0.683 | 99 | 80 | 51 | Precuneus | medial area 5 |
| A4ll_r | 0.683 | 96 | 105 | 48 | Paracentral Lobule | area 4 |
| A4ll_l | 0.610 | 87 | 104 | 48 | Paracentral Lobule | area 4 |
| A7m_r | 0.589 | 97 | 62 | 59 | Precuneus | medial area 7 |
| A7m_l | 0.589 | 86 | 63 | 147 | Precuneus | medial area 7 |
| A23c_l | 0.551 | 84 | 104 | 68 | Cingulate Gyrus | caudal area 23 |
| A6m_l | 0.491 | 85 | 122 | 51 | Superior Frontal Gyrus | medial area 6 |
| A24cd_r | 0.472 | 95 | 133 | 70 | Cingulate Gyrus | caudodorsal area 24 |
| A6m_r | 0.462 | 99 | 123 | 49 | Superior Frontal Gyrus | medial area 6 |
| vmPOS_r | 0.452 | 105 | 64 | 98 | Medio Ventral Occipital Cortex | ventomeial parietooccipital sulcus |
| TH_r | 0.404 | 109 | 92 | 121 | Parahippocampal Gyrus | area TH |
| **Component #10: Thalamus** | | | | | | |
| Stha_r | 0.742 | 109 | 106 | 106 | Thalamus | sensory thalamus |
| mPMtha_r | 0.709 | 104 | 114 | 108 | Thalamus | pre-motor thalamus |
| IPFtha_l | 0.582 | 80 | 113 | 106 | Thalamus | lateral pre-frontal thalamus |
| Stha_l | 0.501 | 73 | 105 | 105 | Thalamus | sensory thalamus |
| A44op_l | 0.488 | 52 | 151 | 104 | Inferior Frontal Gyrus | opercular area 44 |
| vmPu_l | 0.429 | 68 | 135 | 112 | Basal Ganglia | ventromedial putamen |
| cHipp_r | 0.422 | 120 | 101 | 120 | Hippocampus | caudal hippocampus |
| Otha_l | 0.419 | 76 | 100 | 105 | Thalamus | occipital thalamus |
| **Component #11: Medial superior frontal gyrus/anterior cingulate cortex/orbital gyrus** | | | | | | |
| A32sg_r | 0.748 | 96 | 169 | 101 | Cingulate Gyrus | subgenual area 32 |
| A14m_r | 0.687 | 97 | 176 | 114 | Orbital Gyrus | medial area 14 |
| A14m_l | 0.684 | 84 | 182 | 114 | Orbital Gyrus | medial area 14 |
| A10m_r | 0.663 | 99 | 186 | 94 | Superior Frontal Gyrus | medial area 10 |
| A9m_l | 0.626 | 87 | 163 | 69 | Superior Frontal Gyrus | medial area 9 |
| A9m_r | 0.601 | 97 | 165 | 73 | Superior Frontal Gyrus | medial area 9 |
| A11m_r | 0.582 | 98 | 185 | 124 | Orbital Gyrus | medial area 11 |
| A32p_l | 0.578 | 85 | 162 | 87 | Cingulate Gyrus | pregenual area 32 |
| A32p_r | 0.564 | 96 | 155 | 80 | Cingulate Gyrus | pregenual area 32 |
| A45c_r | 0.545 | 145 | 151 | 97 | Inferior Frontal Gyrus | caudal area 45 |
| A32sg_l | 0.508 | 87 | 167 | 110 | Cingulate Gyrus | subgenual area 32 |
| A45r_r | 0.488 | 142 | 164 | 108 | Inferior Frontal Gyrus | rostral area 45 |
| A22r_l | 0.466 | 36 | 125 | 118 | Superior Temporal Gyrus | rostral area 22 |
| A1_2_3tonIa_r | 0.456 | 147 | 117 | 94 | Postcentral Gyrus | area 1/2/3 |
| A12_47l_r | 0.448 | 133 | 159 | 117 | Orbital Gyrus | lateral area 12/47 |
| A24cd_r | 0.449 | 95 | 133 | 70 | Cingulate Gyrus | caudodorsal area 24 |
| **Component #12: Medio ventral occipital cortex** | | | | | | |
| cCunG_l | 0.593 | 85 | 34 | 110 | Medio Ventral Occipital Cortex | caudal cuneus gyrus |
| cCunG_r | 0.555 | 99 | 38 | 98 | Medio Ventral Occipital Cortex | caudal cuneus gyrus |
| msOccG_l | 0.513 | 80 | 39 | 80 | Lateral Occipital Cortex | medial superior occipital gyrus |
| msOccG_r | 0.490 | 107 | 42 | 76 | Lateral Occipital Cortex | medial superior occipital gyrus |
| rcunG_r | 0.448 | 98 | 52 | 99 | Medio Ventral Occipital Cortex | rostral cuneus gyrus |
| **Component #13:** | | | | | | |
| dlPu_l | 0.666 | 63 | 123 | 107 | Basal Ganglia | dorsolateral putamen |
| vmPu_l | 0.637 | 68 | 135 | 112 | Basal Ganglia | ventromedial putamen |
| A24rv_r | 0.512 | 96 | 149 | 96 | Cingulate Gyrus | rostroventral area 24 |
| vIa_l | 0.480 | 59 | 142 | 121 | Insular Gyrus | ventral agranular insular |
| lAmyg_l | 0.447 | 64 | 124 | 128 | Amygdala | lateral amygdala |
| NAC_l | 0.426 | 74 | 132 | 118 | Basal Ganglia | nucleus accumbens |
